# Evaluation of Orofacial and General Pain Location in Patients With Temporomandibular Joint Disorder—Myofascial Pain With Referral

**DOI:** 10.3389/fneur.2019.00546

**Published:** 2019-05-29

**Authors:** Joanna Kuć, Krzysztof Dariusz Szarejko, Teresa Sierpińska

**Affiliations:** ^1^Department of Prosthodontics, Medical University of Bialystok, Bialystok, Poland; ^2^Private Health Care, Physical Therapy and Rehabilitation, Bialystok, Poland

**Keywords:** orofacial pain, myofascial pain, referred pain, temporomandibular disorder, headache

## Abstract

**Introduction:** Pain is an emotional experience. As a subjective feeling, it is associated with pathophysiological processes occurring in the central nervous system, which in turn may negatively affect the psychophysical function, cognitive abilities, level of functioning and quality of life.

**The Aim:** The aim of the study was to assess orofacial and general pain location in patients with temporomandibular joint disorder—myofascial pain with referral.

**Materials and Methods:** The study group consisted of 50 randomly selected, generally healthy people with complete natural dentition (37 women and 13 men) at the age of 23.36 ± 2.14 years, referred to the Department of Prosthodontics of the Medical University. All patients underwent clinical examination according to the Diagnostic Criteria for Temporomandibular Disorders (Axes I and II). The subjects were classified as people with myofascial pain with referral. The evaluation of severity of temporomandibular disorders was based on the Temporomandibular Disorder Pain Screener and the Graded Chronic Pain Scale. In order to assess orofacial and general pain location, a bodychart drawing of pain was used.

**Results:** The study group indicated 40 different areas of the body affected by pain. 2–3 isolated pain locations were declared by a total of six subjects. One person identified 17 affected areas. Forty four people reported pain in at least four regions of the body. 70% of patients suffered from pain within the right masseter muscle. Pain of the left masseter muscle was noted in 68% of cases. Cervical ailments were reported by 56% of people. Pain of the left temporomandibular joint was observed in 68% of patients, and of the right one in 54%.

**Conclusion:** The patients with myofascial pain with referral suffer from general ailments in different regions of the body. Only the frequency of pain in the right masseter muscle and right temporomandibular joint differed with respect to gender. The suggestion that the prevalence of pain in other areas of the body varies between men and women has not been confirmed. Due to a small sample size, such differences cannot be excluded. Further studies in this area are needed.

## Introduction

Functional disorders of temporomandibular joints belong to the group of chronic facial disorders and affect about 10–15% of the total population (1). Women suffer twice as often as men (1). The most common type of dysfunction is myalgia, which intensifies during daily activities and muscle palpation. It is characterized by the occurrence of headache, referred pain, and the restriction of mandible mobility (1). A possible cause is excessive teeth clenching, which leads to disturbances in local muscle blood flow and consequently results in ischemia (1). It promotes the secretion of bradykinin, protons, serotonin, glutamate, or cytokines that sensitize nociceptors, causing muscle pain and/or allodynia (1–3). Repetitive parafunctional activity through temporal summation maintains chronic muscle pain (1, 4). An increased concentration of biomarkers such as IL-1ß, IL-6, IL-7, IL-8, IL-10, IL-13, TNF, and IL-1ra is observed (2, 5–7).

According to the definition of The International Study of Pain, pain is defined as an unpleasant sensory and emotional experience related to real or potential tissue damage or is described in terms of such damage (8, 9). Pain is a subjective feeling. Due to the unpleasant impression, it affects emotional experience (8, 9). Chronic pain lasts longer than the healing of the damaged tissue and is associated with pathophysiological processes which occurs in the central nervous system, which in turn may negatively affect the emotional state and psychophysical function, cognitive abilities, level of functioning, and quality of life. Chronic pain is defined as continuous or recurrent and lasting for more than 3–6 months (9). The options for treatment of chronic pain include pharmacological agents, surgical procedures, psychological therapies, rehabilitation, physiotherapy, as well as alternative medicine (9). Pharmacological treatment is applied in accordance with the criteria of the WHO (World Health Organization) analgesic ladder. Aspirin, non-steroidal anti-inflammatory drugs and opioids are recommended (9). Alternative therapies include massage, yoga, chiropractic, acupuncture, and magnetotherapy (9).

The aim of the study was to assess orofacial and general pain location in patients with temporomandibular joint disorder—myofascial pain with referral. The hypothesis was that the prevalence of pain in different areas of the body varies between men and women.

## Materials and Methods

### The Subjects and Sample Size

The study group consisted of 50 randomly selected, generally healthy Caucasian people (37 women and 13 men) at the age of 23.36 ± 2.14 years (women: mean 23.19 ± 2.31, Me = 24; men: mean 23.85 ± 1.57, Me = 24), referred to the Department of Prosthodontics of the Medical University. All the participants were in the process of obtaining higher education, had never married and had at least good household income. The qualification criterion was the presence of pain in the cranio-facial and/or cranio-mandibular area at the level of 8 points in the VAS (Visual Analog Scale) on clinical examination. The evaluation was performed by a researcher who was also a dentist and physiotherapist. The patients represented complete natural dentition with the intercuspation corresponding to Class I, according to Angle, with no history of orthodontic treatment or retention status after its completion exceeding 3 years. Regarding the DC/TMD (Diagnostic Criteria for Temporomandibular Disorders), the subjects were classified as suffering from myofascial pain with referral pain (10–13). Sixty seven out of 100 examined temporomandibular joints had no symptoms of dysfunction with respect to the DC/TMD. In 30 cases, disc dislocation with a reduction was found, and in another three, one disc dislocation with reduction and intermittent locking was observed.

People who had previous traumas and surgical procedures in the craniofacial area were excluded from participation. Cases affected by metabolic diseases and people whose medication or possible ailments could influence the functioning of masticatory muscles were also excluded. The group did not declare a history of physiotherapeutic treatment in the cranio-facial, cranio-mandibular, and/or cranio-cervical areas.

### Clinical Procedure

**Figure 1:**
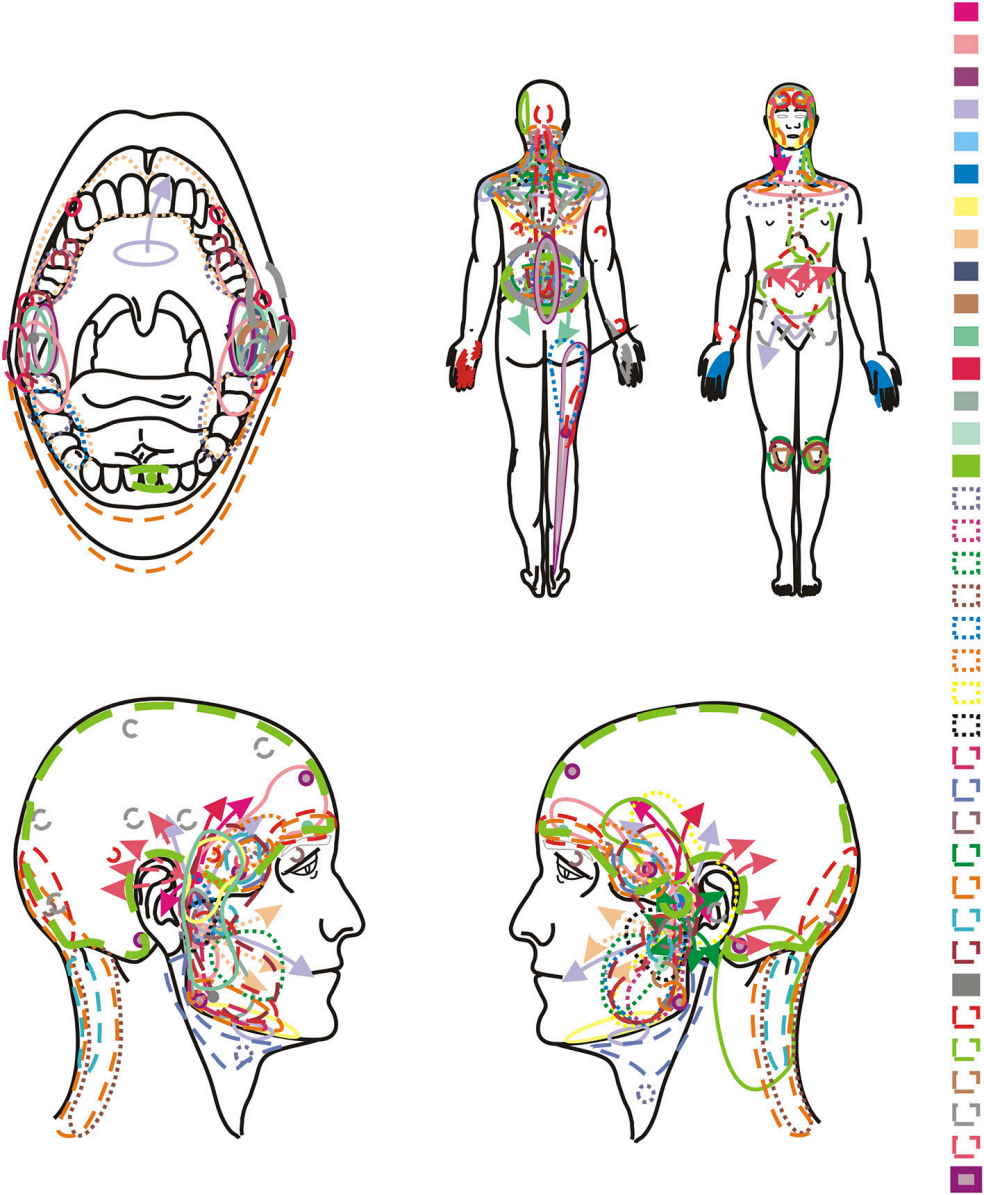
Orofacial and general pain location in group of women (*n* = 37).

**Figure 2:**
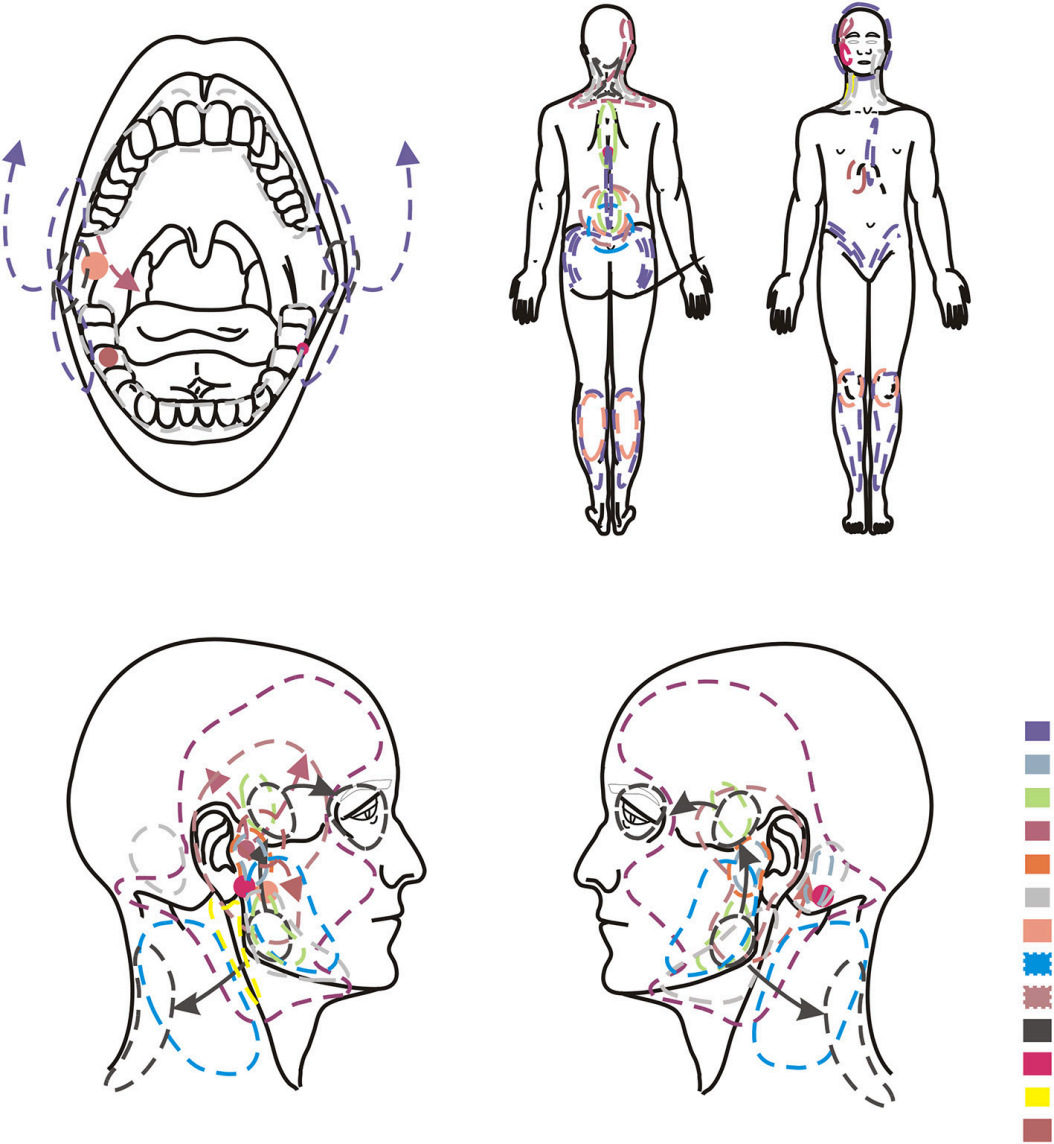
Orofacial and general pain location in group of men (*n* = 13).

All patients underwent a thorough assessment. The proceedings covered:

- Clinical examination including functional evaluation of temporomandibular joints and muscles of the masticatory system according to the DC/TMD (10– 13)—axis I
- TMD Pain Screener—axis I of the DC/TMD (Supplementary Material)
- Graded Chronic Pain Scale version 2.0—axis II of the DC/TMD (Supplementary Material)
- Pain drawing (Bodychart) (Figures 1, 2) to assess orofacial and general pain location—axis II of the DC/TMD. The patients were asked to mark the sites of all pain in the body. In the case of localized pain, “•” mark was used. If the pain changed, then arrows were used to indicate how the pain location moved.

### Statistical Analysis

Statistical analysis was carried out using Statistica 12 Software (StatSoft Power Solutions, Inc.) (14) (Supplementary Material). A Chi-square test of independence for 2 × 2 table was calculated comparing the frequency of pain locations in men and women. In the cases of small samples (expected number of frequencies fewer than 5), Fisher's Exact one-tailed test was additionally used. Differences in *p* < 0.05 were considered as statistically significant. With respect to Fisher's exact test, statistical post hoc power analysis was performed using G Power v. 3.1.9.4 Software (Germany). Power (1-ß) was calculated as the function of α, the population effect size and N.

### Ethical Approval

The project was carried out after obtaining consent from the Bioethical Commission of the Medical University No R-I-002/322/2016. The research was performed in accordance with the Helsinki Declaration of the World Association of Physicians and the principles of Correct Clinical Trial Guidance (Guidelines for Good Clinical Practice). Participation in the project was voluntary. Patients had obtained comprehensive information about the nature, scope of clinical activities and the course of the proceedings. At each stage, the respondents had the right to refuse to participate in the study, without any corresponding consequences. Participation in the study was preceded by the patient's written informed consent.

## Results

The study involved 50 patients, 13 men and 37 women. Seventy percent of the people reported a possible occurrence of functional disorders of temporomandibular joints (TMD-PSc = 4–6) (Table 1). In 15 people (TMD-PSc = 0–3), including 11 women and 4 men, the presence of dysfunction was dubious (Table 1). The prevalence of temporomandibular joint disorders in the group of women and men was comparable, at 70%.

**Table 1:** TMD-Pain Screener results (Axis I of DC/TMD) in the whole study group (*n* = 50), group of women (*n* = 37), and men (*n* = 13).

**TMD Pain screener**	**Reference value**	**Whole study group *n* = 50**	**Group of women *n* = 37**	**Group of men *n* = 13**
Dubious presence of TMD	0–3	15 (30.00%)	11 (29.73%)	4 (30.77%)
The potential presence of TMD	4–6	35 (70.00%)	26 (70.27%)	9 (69.23%)

*The number of people (n) and percentages (%) are given*.

In three patients from the study group, no significant chronic TMJ pain was found in the last 6 months with respect to GCPS v.2. (Table 2). 30 (60%) of the subjects displayed low intensity of pain without functional disorders. High intensity of pain and low disability (II°) or moderate limitation (III°), was reported by six (12%) patients. In five (10%) subjects, high disability with severe limitation was found (Table 2).

**Table 2:** Graded Chronic Pain Scale v.2.

**GCPS v.2**.	**Description**	**Whole study group *n* = 50**	**Group of women *n* = 37**	**Group of men *n* = 13**
Grade 0	No TMJ pain in the last 6 months	3 (6%)	2 (5%)	1 (8%)
Grade I	Low intensity of pain	30 (60%)	24 (65%)	6 (46%)
	Low disability			
Grade II	High intensity of pain	6 (12%)	4 (11%)	2 (15%)
	Low disability			
Grade III	High Disability	6 (12%)	2 (5%)	4 (31%)
	Moderately Limiting			
Grade IV	High Disability	5 (10%)	5 (14%)	0 (0%)
	Severely Limiting			

*(GCPS v.2.) results in the whole study group (n = 50), group of women (n = 37) and men (n = 13). The number of people (n) and percentages (%) are given*.

Seventy percent of patients suffered from pain within the right masseter muscle. Pain in the left masseter muscle was noted in 68% of cases. Cervical ailments were reported by 56% of participants. Pain of the temporomandibular joint was observed in 68% of patients on the left side and in 54% on the right side (Table 3).

**Table 3:** Orofacial and general pain distribution with respect to the bodychart (pain drawing) in the whole study group (*n* = 50), group of women (*n* = 37), and men (*n* = 13).

**Area of the body**	**The number of people (*n*) and percentages (%) with respect to the whole study group (*n* = 50)**	**The number of people (*n*) and percentages (%) with respect to the group of women (*n* = 37)**	**The number of people (*n*) and percentages (%) with respect to the group of men (*n* = 13)**	**Chi**^**2**^ **Pearsons**			**Fisher's Exact Unilateral Test**	**with respect to Fisher's Exact Unilateral Test**
				**Chi^2^**	**df**	***p =***	***p =***	**Power (1-ß)**
Cervical spine Cx	28 (56.00%)	23 (62.16%)	5 (38.46%)	2.193077	1	*P* = 0.13863	*P* = 0.12404	0.3096000
Thoracic spine Tx	24 (48.00%)	16 (43.24%)	8 (61.54%)	1.290047	1	*P* = 0.25604	*p* = 0.20828	0.1962761
Lumbar spine Lx	24 (48.00%)	18 (48.65%)	6 (46.15%)	0.0239885	1	p = 0.87691	*p =* 0.56717	0.0336837
Sacrum Sc	9 (18.00%)	6 (16.22%)	3 (23.08%)	0.3067796	1	*p =* 0.57966	*p =* 0.42950	0.0781242
Pelvis	5 (10.00%)	5 (13.51%)	0 (00%)	1.951952	1	*p =* 0.16238	*p =* 0.20573	0.0539917
Temporal muscle on the right side	26 (52.00%)	20 (54.05%)	6 (46.15%)	0.2405512	1	*p =* 0.62381	*p =* 0.43283	0.0670719
Temporal muscle on the left side	24 (48.00%)	20 (54.05%)	4 (30.77%)	2.089664	1	*p =* 0.14830	*p =* 0.13055	0.2965980
Masseter muscle on the right side	35 (70.00%)	23 (62.16%)	12 (92.31%)	4.162954	1	*p =* 0.04132^*^	*p =* 0.03943 ^*^	0.5351020
Masseter muscle on the left side	34 (68.00%)	26 (70.27%)	8 (61.54%)	0.3370735	1	*p =* 0.56152	*p =* 0.39997	0.0841976
Musculus sternocleidomastoideus on the right side	6 (12.00%)	3 (8.11%)	3 (23.08%)	2,041202	1	*p =* 0.15309	*p =* 0.17292	0.2675022
Musculus sternocleidomastoideus on the left side	7 (14.00%)	3 (8.11%)	4 (30.77%)	4.103094	1	*p =* 0.04280^*^	*p =* 0.06485	0.4687233
TMJ on the left side	34 (68.00%)	27 (72.97%)	7 (53.85%)	1.617341	1	*p =* 0.20346	*p =* 0.17649	0.2405843
TMJ on the right side	27 (54.00%)	16 (43.24%)	11 (84.62%)	6.628870	1	*p =* 0.01003^*^	*p =* 0.01048^*^	0.7871910
Area of the lower angle of the left scapula	4 (8.00%)	4 (10,81%)	0 (0.00%)	1.527615	1	*p =* 0.21647	*p =* 0.28678	0.0150241
Area of the lower angle of the right scapula	4 (8.00%)	4 (10.81%)	0 (0.00%)	1.527615	1	*p =* 0.21647	*p =* 0.28678	0.0150241
Right shoulder	16 (32.00%)	13 (35.14%)	3 (23.08%)	0.6428091	1	*p =* 0.42270	*p =* 0.33102	0.1141421
Left shoulder	16 (32.00%)	14 (37.84%)	2 (15.38%)	2.228812	1	*p =* 0.13546	*p =* 0.12409	0.3233793
Thoracic outlet	3 (6.00%)	3 (8.11%)	0 (0.00%)	1,121334	1	*p =* 0.28963	*p =* 0.39643	0.0023215
Right hip band	2 (4.00%)	2 (5.41%)	0 (0.00%)	0.7319820	1	*p =* 0.39224	*p =* 0.54367	0.0001234
Left hip band	1 (2.00%)	1 (2.70%)	0 (0.00%)	0.3585218	1	*p =* 0.54933	*p =* 0.74000	0.0000000
Sternum	3 (6.00%)	2 (5.41%)	1 (7.69%)	0.0892054	1	*p =* 0.76519	*p =* 0.60357	0.0334427
Right knee	6 (12.00%)	4 (10.81%)	2 (15.38%)	0.1905752	1	*p =* 0.66244	*p =* 0.49710	0.0614422
Left knee	6 (12.00%)	4 (10.81%)	2 (15.38%)	0.1905752	1	*p =* 0.66244	*p =* 0.49710	0.0614422
Right shinbone	4 (8.00%)	2 (5.41%)	2 (15.38%)	1.301636	1	*p =* 0.25391	*p =* 0.27462	0.1743806
Left shinbone	3 (6.00%)	1 (2.70%)	2 (15.38%)	2.743251	1	*p =* 0.09767	*p =* 0.16793	0.2638459
Teeth 18-14	6 (12.00%)	5 (13.51%)	1 (7.69%)	0.3087003	1	*p =* 0.57848	*p =* 0.50290	0.0191525
Teeth 13-11	2 (4.00%)	1 (2.70%)	1 (7.69%)	0.6237006	1	*p =* 0.42968	*p =* 0.45633	0.0562780
Teeth 21-23	2 (4.00%)	1 (2.70%)	1 (7.69%)	0.6237006	1	*p =* 0.42968	*p =* 0.45633	0.0562780
Teeth 24-28	6 (12.00%)	5 (13.51%)	1 (7.69%)	0.3087003	1	*p =* 0.57848	*p =* 0.50290	0.0191525
Teeth 34-38	6 (12.00%)	4 (10.81%)	2 (15.38%)	0.1905752	1	*p =* 0.66244	*p =* 0.49710	0.0614422
Teeth 31-33	3 (6.00%)	2 (5.41%)	1 (7.69%)	0.0892054	1	*p =* 0.76519	*p =* 0.60357	0.0334427
Teeth 41-43	4 (8.00%)	3 (8.11%)	1 (7.69%)	0.0022598	1	*p =* 0.96209	*p =* 0.72538	0.0008206
Teeth 44-48	7 (14.00%)	5 (13.51%)	2 (15.38%)	0.0279733	1	*p =* 0.86717	*p =* 0.59435	0.0350405
Palate	1 (2.00%)	1 (2.70%)	0 (0.00%)	0.3585218	1	*p =* 0.54933	*p =* 0.74000	0.0000000
Right hand	1 (2.00%)	1 (2.70%)	0 (0.00%)	0.3585218	1	*p =* 0.54933	*p =* 0.74000	0.0000000
Left hand	2 (4.00%)	2 (5.41%)	0 (0.00%)	0.7319820	1	*p =* 0.39224	*p =* 0.54367	0.0001234
Right pterygomandibular ligament	8 (16.00%)	6 (16.22%)	2 (15.38%)	0.0049500	1	*p =* 0.94391	*p =* 0.65896	0.0156287
Left pterygomandibular ligament	11 (22.00%)	10 (27.03%)	1 (7.69%)	2.095721	1	*p =* 0.14771	*p =* 0.14412	0.2889457
Right eye	1 (2.00%)	0 (0.00%)	1 (7.69%)	0.3585218	1	*p =* 0.54933	*p =* 0.74000	0.0726438
Left eye	1 (2.00%)	0 (0.00%)	1 (7.69%)	0.3585218	1	*p =* 0.54933	*p =* 0.74000	0.0726438

*The number of people (n) and percentages (%) are given*.

* *p < 0.05 statistical significance*.

With respect to gender, a statistically significant difference in the prevalence of pain was noted within the right masseter muscle (χ^2^ = 4.162954, *p* = 0.04132) (Table 3). Test's power to detect the specified effect was on the medium level (Fisher's Exact Unilateral Test: *p* = 0.03943, 1-ß = 0.5351020) (Table 3). 92.31% of men and 62.16% of women suffered from pain in this area (Table 3). A similar tendency was found with regard to the right TMJ (χ^2^ = 6.628870, *p* = 0.01003). In this case, the test's power was slightly higher (Fisher's Exact Unilateral Test: *p* = 0.01048, 1-ß = 0.7871910). 84.62% of men and 43.24% of women suffered from pain of the right temporomandibular joint (Table 3). With regards to other areas of the body, the test's power to detect the specified effect was low (1-β < 0.5) (Table 3).

**Figure 3:**
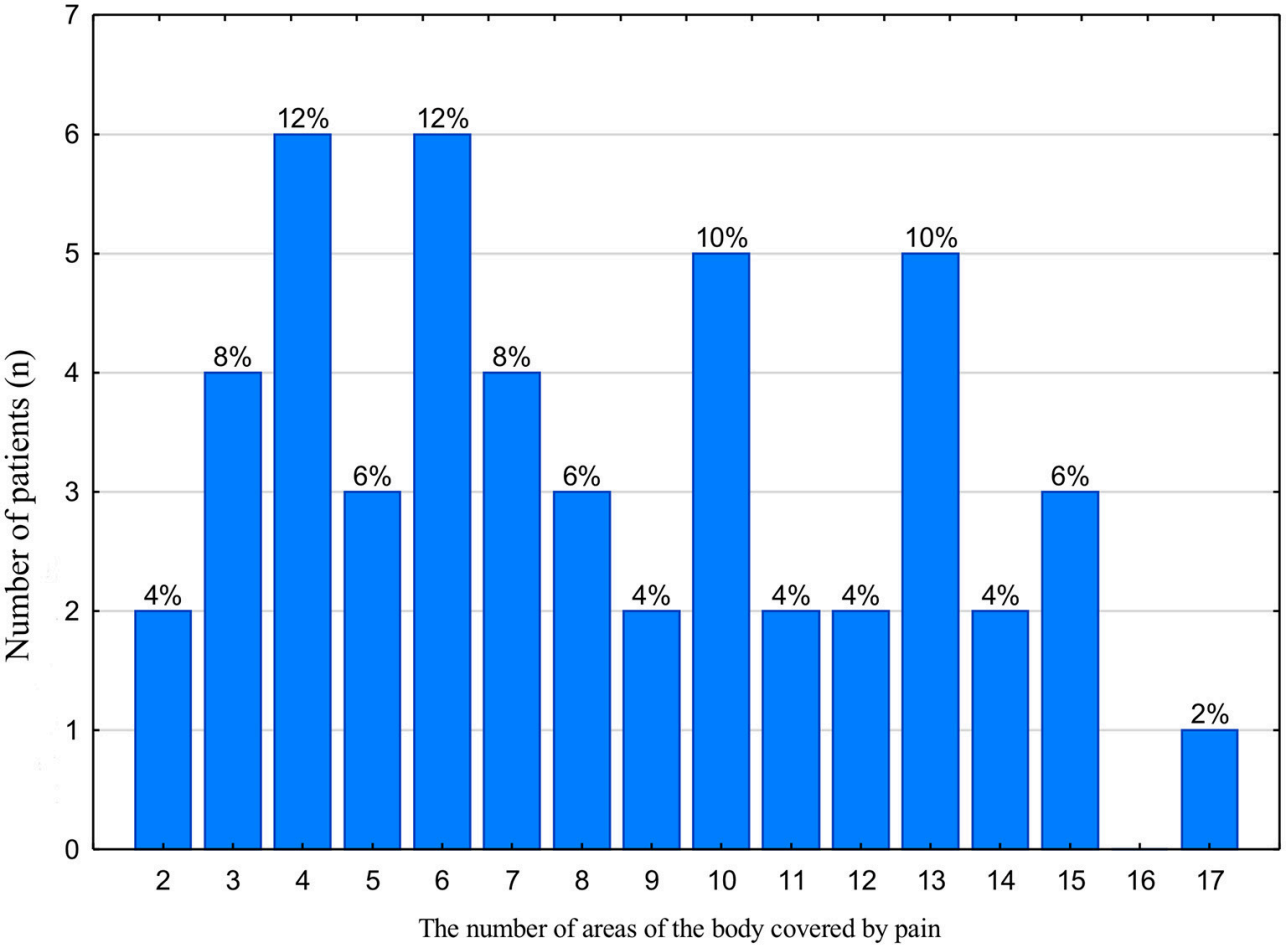
Location of the pain areas in the whole study group (*n* = 50). The number of patients and the corresponding percentage in the study group are given.

The study group indicated 40 different areas of the body affected by pain (Figure 3). Up to three isolated pain locations were declared by a total of six subjects. One person identified 17 affected areas (Figure 3). Forty four people reported pain in at least four regions of the body.

## Discussion

The incidence of specific types of temporomandibular joint disorders with respect to the DC/TMD classification, depending on the studied population, is variable. John et al. stated that in a group of 416 women, 27.4% were cases of myofascial pain, 21.4% subjects had myofascial pain associated with mobility restriction of the mandible, 44.2% were patients with dislocation of the disc with the possibility of reduction, and 6.3% cases were the displacement of the disc without reduction (15). Arthralgia was found in 33.2% of cases, osteoarthritis in 3.6%, and osteoarthritis in 3.4%. Many patients had more than one diagnosis (15). On the other hand, a study carried out among Swedish dentistry students without overt dysfunctions revealed the occurrence of disorders in 30% of the subjects. According to the DC/TMD criteria, the most frequent pathologies were disorders from the myalgia group (16).

In the presented study it was found that the potential presence of functional disorders of temporomandibular joints can affect up to 70% of patients, including 26 women and 9 men (Table 1). The remaining 15 people displayed dubious results. The study revealed a broad spectrum of disorders of the II axis of the DC/TMD protocol in relation to the bodychart (pain drawing) (Figures 1, 2). The importance of the biopsychosocial component in the assessment of temporomandibular joint disorders, including myofascial dysfunction, was suggested.

The results of this study obtained on the basis of the bodychart reflect the characteristic profile of myofascial disorders associated with referred pain (Table 3, Figures 1, 2). Attention was paid to the multifaceted nature of the ailments indicated by the respondents, as well as a typical pattern of transfer from trigger points (Figures 1, 2). Suvinen et al. reported that extensive pain is related to a higher risk of depression and somatization, reduced levels of overall health status, increased propensity to sleep disorders, decreased pain control capacity, and increased healthcare needs compared to patients with localized pain (17). In the group of 135 people examined by the above-mentioned author, 21% patients suffered from local myalgia and 20% declared pain limited to the examined body area within the head and neck (1). Fifty nine percent of the respondents reported generalized pain covering many zones of the body: 28.2% declared severe and 30.8% moderate disability determined by their complaints (17).

In our study, the patients were a homogeneous group of people with myofascial pain with pain referral. With regard to the bodychart, only two people indicated ailments limited to two places of occurrence (Figure 3). Forty eight subjects declared the presence of pain in three or more zones, which suggests serious pathologies and raises the risk of developing systemic disorders based on central sensitization of pain, which promotes the possibility of chronic pain (12, 18). In these cases, the need for general treatment should be considered with respect to the DC/TMD recommendations (12).

Particular attention should be paid to pain in the cervical, thoracic and lumbar spine observed in ± 50% of the subjects (Table 3) (Figures 1, 2). The obtained results may indicate co-occurring postural abnormalities, bad habits, poor ergonomics in everyday activities, and the resultant need for postural reeducation. The direct binding factor for the masticatory dysfunction is undoubtedly upper cervical spine disorders. This is dictated by both the anatomophysiological aspects of the C0-C2 complex (Occiput-Axis), including a neurological component (C2 nerve root), and often also traumatic etiology of whiplash related to this segment. An injury contributes to the temporomandibular joint disorder directly or through a delayed response, most often controlled by means of central sensitization of pain (19). This promotes the transition of the acute phase into a chronic one, thereby initiating the occurrence of chronic pain (19).

Bogduk et al. emphasize the role of convergence between cervical and trigeminal afferents in the trigeminocervical nucleus. This author indicates that nociceptive afferents from C1, C2, and C3 spinal nerves converge with the first division of the trigeminal nerve, which mediates referred pain from the neck to the head (to occipital, auricular, parietal and orbital regions) (20).

Pain from the cervical zygapophysial joints, which has constant segment patterns, must also be mentioned. Pain within zygapophysial joints at the C2-C3 level is referred toward the front of the head. Pain from C3-C4 and C4-C5 remains within the posterior part of the neck. A typical location of spreading pain from C5-C6 is supraspinous fossa of the scapula. C6-C7 generates spreading pain caudally over the scapula (21).

Pedroni et al. noted that most frequently the pain area indicated by TMD patients was the cervical spine (92.85%) and scapular region (50%). The third most commonly observed location was TMJ (42.85%), followed by masseter muscle (35.71%), temporal muscle (21.42%), and frontal region (28.57%) (22).

Wright et al. indicated the significance of postural re-education in reducing pain in temporomandibular joints, as well as in attempts to improve the extent of mouth opening (23). Komiyama et al. also emphasize the importance of postural correction in the treatment of patients with myofascial pain with reduced mobility of the mandible (24). Other reports support the positive effect of postural exercises as well as active and passive exercises directed to the lower jaw and the cervical spine (25).

Based on the presented results, the bodychart appears to be an extremely useful diagnostic screening tool. It constitutes a part of the comprehensive biopsychosocial assessment as well as the way of programming therapy in patients with temporomandibular joint disorder (17).

According to the chronic pain scale GCPS v 2.0, grade I of complaints was observed in 60% of the subjects (Table 2). In the case of grades II and III of disorders, 12% results were recorded in each group. A severe functional limitation was found in 10% of patients. Manfredini et al. emphasize the fact that current research on chronic pain in patients with masticatory dysfunction indicates that the mean incidence in grades I and II of disorders in relation to the GCPS v 2.0 scale is 35–40%, for grade III, 15–18%, and in grade IV it reaches 3–6% (26). This author indicates that the first research on the second axis of the DC/TMD protocol noted a strong relationship between GCPS and somatization as well as weak links with levels of depression (27). On the other hand, multicenter data from more representative samples indicate an important relationship between somatization, depression and GCPS, thus supporting the early view that the three main elements of the second axis of the protocol are closely related (28).

It is also interesting to note that in the case of patients with musculoskeletal pain and temporomandibular joint disorders, chronic pain is the cause of limited activity in everyday life, as well as psychosocial dysfunction (29–32). At the moment, in the case of chronic pain assessment, the interval time of existing ailments is binding. The main criterion is the presence of symptoms lasting for over 3 or 6 months (25). Manfredini et al. additionally point to the essence of qualitative features of chronic pain, i.e., durability, intensity or fluctuations, and the significance of conditions related to emotional anxiety or being the cause of a lack of instruction (26, 33).

The results obtained with regard to GCPS v 2.0 differed from those listed in the literature and are most likely determined by the homogeneity of the study group, only including cases of myofascial pain with referral. According to Reiter et al., study results are often conditioned by the social context, ethnic origin, culture, personality traits, as well as the level of intelligence (34).

In turn, in the studies by Olivo et al. carried out in a group of 45 women aged 18–50 years old with temporomandibular joints dysfunction with myogenic etiology, grade I of chronic pain intensity was found in 19 people with respect to the GCPS scale, grades II and IV only in one case, respectively, and grade III in 24 subjects (35). On the other hand, in a mixed group including both myofascial disorders of the craniofacial region and temporomandibular joint disorders, there were 12 cases in grade I of chronic pain severity, three people in grade II, 22 patients in grade III and seven in grade IV out of 44 people affected by the dysfunction (35).

Our study was designed to induce reflection of clinicians treating patients with temporomandibular joint disorders. Pain locations indicated the multifaceted nature of complaints in people who potentially declared good health. The bodychart revealed the size of the patients' problems. Many areas of complaints may suggest processing disorders and central sensitization. Pain drawings emphasized the essence of the biopsychological component in this group of patients and the need to cooperate in an interdisciplinary team.

### Strengths and Limitations of the Study

Standardized procedures (DC/TMD protocol) allow the study to be repeated in similar research projects with the observation of comparable findings. Clear documentation of “Pain Drawing” allows other researchers using the DC/TMD protocol to assess the validity of the study results. The use of the Pain Drawing (DC/TMD protocol) emphasizes the need for holistic treatment in patients with craniofacial disorders. The bodychart reflects the specific profile of myofascial pain with referral. It is possible to estimate the cost and benefits of clinical prosthetic and physiotherapeutic procedures. In each case it is possible to determine the possible pattern of descending or ascending hereto or unilateral disorders corresponding to the craniomandibular dysfunction.

Self-reported information obtained from the bodychart may be inaccurate or incomplete. The clinical protocol for examining patients in accordance with the guidelines of DC/TMD requires precision and is time-consuming. Due to extensive DC/TMD instruments, it is not possible to present all data in one study, which may result in the omission of information that is key for the subject's case. Some information is difficult to receive through DC/TMD protocol, particularly on sensitive topics such as role of dura mater in TMJ disorders. The DC/TMD protocol does not include a clinical examination of many muscles affecting the mobility of the mandible. Interdisciplinary cooperation with physiotherapists is necessary. Research methods are inflexible, and the protocol is imposed in advance. The expanded DC/TMD questionnaire (Axes I and II) may alienate respondents. The bodychart results may mask or ignore underlying structural causes or sources of pain. Due to the small research group, further studies in this area are needed.

## Conclusion

The bodychart is an effective research and clinical tool which allows one to reflect unconscious pain. The patients with myofascial pain with referral suffer from general ailments in different regions of the body. Only the frequency of pain of the right masseter muscle and right temporomandibular joint differed with respect to gender. The suggestion that the prevalence of pain in other areas of the body varies between men and women has not been confirmed. Due to the small sample size, such differences cannot be excluded. Further studies in this area are needed.

## Ethics Statement

The project was carried out after obtaining the consent of the Bioethical Commission of the Medical University of Białystok No R-I-002/322/2016. The research was performed in accordance with the Helsinki Declaration of the World Association of Physicians and the principles of Correct Clinical Trial Guidance (Guidelines for Good Clinical Practice). Participation in the project was voluntary. Patients had obtained comprehensive information about the nature, scope of clinical activities and the course of the proceedings. At each stage, the respondents had the right to refuse to participate in the study, without any corresponding consequences. Participation in the study was preceded by a patient's written consent.

## Author Contributions

JK conceived and planned the study. JK and KS carried out the experiment. JK and KS contributed to sample preparation. TS contributed to the interpretation of the results. JK and KS took the lead in writing the manuscript. TS supervised the project. All authors discussed the results and contributed to manuscript revision, read and approved the submitted version.

### Conflict of Interest Statement

The authors declare that the research was conducted in the absence of any commercial or financial relationships that could be construed as a potential conflict of interest.
