# Supplementary material for: Evaluation of Orofacial and General Pain Location in Patients With Temporomandibular Joint Disorder—Myofascial Pain With Referral
Source: Front Neurol. 2019 May 29;10:546. doi: 10.3389/fneur.2019.00546 (PMC6549135; doi:10.3389/fneur.2019.00546)
Supplement: Supplementary file 1 [file Data_Sheet_1.docx]

|  | Tabela liczności (Pain drawing statystyka PR-MR .sta)  Tabela:Liczba obszarów ciała(15) x Płeć(2) | | | |
| --- | --- | --- | --- | --- |
|  | Liczba obszarów ciała | Płeć  K | Płeć  M | Wiersz  Razem |
|  | 2 | 2 | 0 | 2 |
|  | 3 | 3 | 1 | 4 |
|  | 4 | 5 | 1 | 6 |
|  | 5 | 1 | 2 | 3 |
|  | 6 | 5 | 1 | 6 |
|  | 7 | 3 | 1 | 4 |
|  | 8 | 1 | 2 | 3 |
|  | 9 | 0 | 2 | 2 |
|  | 10 | 4 | 1 | 5 |
|  | 11 | 2 | 0 | 2 |
|  | 12 | 2 | 0 | 2 |
|  | 13 | 5 | 0 | 5 |
|  | 14 | 2 | 0 | 2 |
|  | 15 | 1 | 2 | 3 |
|  | 17 | 1 | 0 | 1 |
|  | Ogół | 37 | 13 | 50 |

|  | Tabela liczności (Pain drawing statystyka PR-MR .sta)  Tabela:Liczba obszarów ciała(15) x Płeć(2) | | | |
| --- | --- | --- | --- | --- |
|  | Liczba obszarów ciała | Płeć  K | Płeć  M | Wiersz  Razem |
| Liczba | 2 | 2 | 0 | 2 |
| % z kolumny |  | 5,41% | 0,00% |  |
| % z całości |  | 4,00% | 0,00% | 4,00% |
| Liczba | 3 | 3 | 1 | 4 |
| % z kolumny |  | 8,11% | 7,69% |  |
| % z całości |  | 6,00% | 2,00% | 8,00% |
| Liczba | 4 | 5 | 1 | 6 |
| % z kolumny |  | 13,51% | 7,69% |  |
| % z całości |  | 10,00% | 2,00% | 12,00% |
| Liczba | 5 | 1 | 2 | 3 |
| % z kolumny |  | 2,70% | 15,38% |  |
| % z całości |  | 2,00% | 4,00% | 6,00% |
| Liczba | 6 | 5 | 1 | 6 |
| % z kolumny |  | 13,51% | 7,69% |  |
| % z całości |  | 10,00% | 2,00% | 12,00% |
| Liczba | 7 | 3 | 1 | 4 |
| % z kolumny |  | 8,11% | 7,69% |  |
| % z całości |  | 6,00% | 2,00% | 8,00% |
| Liczba | 8 | 1 | 2 | 3 |
| % z kolumny |  | 2,70% | 15,38% |  |
| % z całości |  | 2,00% | 4,00% | 6,00% |
| Liczba | 9 | 0 | 2 | 2 |
| % z kolumny |  | 0,00% | 15,38% |  |
| % z całości |  | 0,00% | 4,00% | 4,00% |
| Liczba | 10 | 4 | 1 | 5 |
| % z kolumny |  | 10,81% | 7,69% |  |
| % z całości |  | 8,00% | 2,00% | 10,00% |
| Liczba | 11 | 2 | 0 | 2 |
| % z kolumny |  | 5,41% | 0,00% |  |
| % z całości |  | 4,00% | 0,00% | 4,00% |
| Liczba | 12 | 2 | 0 | 2 |
| % z kolumny |  | 5,41% | 0,00% |  |
| % z całości |  | 4,00% | 0,00% | 4,00% |
| Liczba | 13 | 5 | 0 | 5 |
| % z kolumny |  | 13,51% | 0,00% |  |
| % z całości |  | 10,00% | 0,00% | 10,00% |
| Liczba | 14 | 2 | 0 | 2 |
| % z kolumny |  | 5,41% | 0,00% |  |
| % z całości |  | 4,00% | 0,00% | 4,00% |
| Liczba | 15 | 1 | 2 | 3 |
| % z kolumny |  | 2,70% | 15,38% |  |
| % z całości |  | 2,00% | 4,00% | 6,00% |
| Liczba | 17 | 1 | 0 | 1 |
| % z kolumny |  | 2,70% | 0,00% |  |
| % z całości |  | 2,00% | 0,00% | 2,00% |
| Liczba | Ogół | 37 | 13 | 50 |
| % z całości |  | 74,00% | 26,00% |  |

| Cx | Podsumowująca tabela dwudzielcza: częstości obserwowane (Pain drawing statystyka PR-MR .sta) | | |
| --- | --- | --- | --- |
|  | Płeć  K | Płeć  M | Wiersz  Razem |
| nie | 14 | 8 | 22 |
| % z kolumny | 37,84% | 61,54% |  |
| % z całości | 28,00% | 16,00% | 44,00% |
| tak | 23 | 5 | 28 |
| % z kolumny | 62,16% | 38,46% |  |
| % z całości | 46,00% | 10,00% | 56,00% |
| Razem w kol. | 37 | 13 | 50 |
| % z całości | 74,00% | 26,00% | 100,00% |

| statystyka | Statystyka: Cx(2) x Płeć(2) (Pain drawing statystyka PR-MR .sta) | | |
| --- | --- | --- | --- |
|  | Chi-kwadr. | df | p |
| Chi^2 Pearsona | 2,193077 | df=1 | p=,13863 |
| Chi^2 NW | 2,188154 | df=1 | p=,13908 |
| Chi^2 Yatesa | 1,336670 | df=1 | p=,24762 |
| dokł. Fishera, 1-stronny |  |  | p=,12404 |
| 2-stronny |  |  | p=,19696 |
| Chi^2 McNemara (A/D) | 3,368421 | df=1 | p=,06646 |
| (B/C) | 6,322581 | df=1 | p=,01192 |

| Tx | Podsumowująca tabela dwudzielcza: częstości obserwowane (Pain drawing statystyka PR-MR .sta) | | |
| --- | --- | --- | --- |
|  | Płeć  K | Płeć  M | Wiersz  Razem |
| nie | 21 | 5 | 26 |
| % z kolumny | 56,76% | 38,46% |  |
| % z całości | 42,00% | 10,00% | 52,00% |
| tak | 16 | 8 | 24 |
| % z kolumny | 43,24% | 61,54% |  |
| % z całości | 32,00% | 16,00% | 48,00% |
| Razem w kol. | 37 | 13 | 50 |
| % z całości | 74,00% | 26,00% | 100,00% |

| statystyka | Statystyka: Tx(2) x Płeć(2) (Pain drawing statystyka PR-MR .sta) | | |
| --- | --- | --- | --- |
|  | Chi-kwadr. | df | p |
| Chi^2 Pearsona | 1,290047 | df=1 | p=,25604 |
| Chi^2 NW | 1,296313 | df=1 | p=,25489 |
| Chi^2 Yatesa | ,6611826 | df=1 | p=,41614 |
| dokł. Fishera, 1-stronny |  |  | p=,20828 |
| 2-stronny |  |  | p=,33883 |
| Chi^2 McNemara (A/D) | 4,965517 | df=1 | p=,02586 |
| (B/C) | 4,761905 | df=1 | p=,02910 |

| Lx | Podsumowująca tabela dwudzielcza: częstości obserwowane (Pain drawing statystyka PR-MR .sta) | | |
| --- | --- | --- | --- |
|  | Płeć  K | Płeć  M | Wiersz  Razem |
| nie | 19 | 7 | 26 |
| % z kolumny | 51,35% | 53,85% |  |
| % z całości | 38,00% | 14,00% | 52,00% |
| tak | 18 | 6 | 24 |
| % z kolumny | 48,65% | 46,15% |  |
| % z całości | 36,00% | 12,00% | 48,00% |
| Razem w kol. | 37 | 13 | 50 |
| % z całości | 74,00% | 26,00% | 100,00% |

| statystyka | Statystyka: Lx(2) x Płeć(2) (Pain drawing statystyka PR-MR .sta) | | |
| --- | --- | --- | --- |
|  | Chi-kwadr. | df | p |
| Chi^2 Pearsona | ,0239885 | df=1 | p=,87691 |
| Chi^2 NW | ,0240081 | df=1 | p=,87686 |
| Chi^2 Yatesa | ,0281532 | df=1 | p=,86675 |
| dokł. Fishera, 1-stronny |  |  | p=,56717 |
| 2-stronny |  |  | p=1,0000 |
| Chi^2 McNemara (A/D) | 5,760000 | df=1 | p=,01640 |
| (B/C) | 4,000000 | df=1 | p=,04550 |

| Sc | Podsumowująca tabela dwudzielcza: częstości obserwowane (Pain drawing statystyka PR-MR .sta) | | |
| --- | --- | --- | --- |
|  | Płeć  K | Płeć  M | Wiersz  Razem |
| nie | 31 | 10 | 41 |
| % z kolumny | 83,78% | 76,92% |  |
| % z całości | 62,00% | 20,00% | 82,00% |
| tak | 6 | 3 | 9 |
| % z kolumny | 16,22% | 23,08% |  |
| % z całości | 12,00% | 6,00% | 18,00% |
| Razem w kol. | 37 | 13 | 50 |
| % z całości | 74,00% | 26,00% | 100,00% |

| statystyka | Statystyka: Sc(2) x Płeć(2) (Pain drawing statystyka PR-MR .sta) | | |
| --- | --- | --- | --- |
|  | Chi-kwadr. | df | p |
| Chi^2 Pearsona | ,3067796 | df=1 | p=,57966 |
| Chi^2 NW | ,2944357 | df=1 | p=,58739 |
| Chi^2 Yatesa | ,0180293 | df=1 | p=,89319 |
| dokł. Fishera, 1-stronny |  |  | p=,42950 |
| 2-stronny |  |  | p=,67948 |
| Chi^2 McNemara (A/D) | 21,44118 | df=1 | p=,00000 |
| (B/C) | ,5625000 | df=1 | p=,45325 |

| Miednica | Podsumowująca tabela dwudzielcza: częstości obserwowane (Pain drawing statystyka PR-MR .sta) | | |
| --- | --- | --- | --- |
|  | Płeć  K | Płeć  M | Wiersz  Razem |
| nie | 32 | 13 | 45 |
| % z kolumny | 86,49% | 100,00% |  |
| % z całości | 64,00% | 26,00% | 90,00% |
| tak | 5 | 0 | 5 |
| % z kolumny | 13,51% | 0,00% |  |
| % z całości | 10,00% | 0,00% | 10,00% |
| Razem w kol. | 37 | 13 | 50 |
| % z całości | 74,00% | 26,00% | 100,00% |

| statystyka | Statystyka: Miednica(2) x Płeć(2) (Pain drawing statystyka PR-MR .sta) | | |
| --- | --- | --- | --- |
|  | Chi-kwadr. | df | p |
| Chi^2 Pearsona | 1,951952 | df=1 | p=,16238 |
| Chi^2 NW | 3,201849 | df=1 | p=,07356 |
| Chi^2 Yatesa | ,7392007 | df=1 | p=,38992 |
| dokł. Fishera, 1-stronny |  |  | p=,20573 |
| 2-stronny |  |  | p=,30873 |
| Chi^2 McNemara (A/D) | 30,03125 | df=1 | p=,00000 |
| (B/C) | 2,722222 | df=1 | p=,09896 |

| Miesien skroniowy prawy | Podsumowująca tabela dwudzielcza: częstości obserwowane (Pain drawing statystyka PR-MR .sta) | | |
| --- | --- | --- | --- |
|  | Płeć  K | Płeć  M | Wiersz  Razem |
| nie | 17 | 7 | 24 |
| % z kolumny | 45,95% | 53,85% |  |
| % z całości | 34,00% | 14,00% | 48,00% |
| tak | 20 | 6 | 26 |
| % z kolumny | 54,05% | 46,15% |  |
| % z całości | 40,00% | 12,00% | 52,00% |
| Razem w kol. | 37 | 13 | 50 |
| % z całości | 74,00% | 26,00% | 100,00% |

| statystyka | Statystyka: Miesien skroniowy prawy(2) x Płeć(2) (Pain drawing statystyka PR-MR .sta) | | |
| --- | --- | --- | --- |
|  | Chi-kwadr. | df | p |
| Chi^2 Pearsona | ,2405512 | df=1 | p=,62381 |
| Chi^2 NW | ,2404882 | df=1 | p=,62385 |
| Chi^2 Yatesa | ,0281532 | df=1 | p=,86675 |
| dokł. Fishera, 1-stronny |  |  | p=,43283 |
| 2-stronny |  |  | p=,75050 |
| Chi^2 McNemara (A/D) | 4,347826 | df=1 | p=,03706 |
| (B/C) | 5,333333 | df=1 | p=,02092 |

| Mięsień skroniowy lewy | Podsumowująca tabela dwudzielcza: częstości obserwowane (Pain drawing statystyka PR-MR .sta) | | |
| --- | --- | --- | --- |
|  | Płeć  K | Płeć  M | Wiersz  Razem |
| nie | 17 | 9 | 26 |
| % z kolumny | 45,95% | 69,23% |  |
| % z całości | 34,00% | 18,00% | 52,00% |
| tak | 20 | 4 | 24 |
| % z kolumny | 54,05% | 30,77% |  |
| % z całości | 40,00% | 8,00% | 48,00% |
| Razem w kol. | 37 | 13 | 50 |
| % z całości | 74,00% | 26,00% | 100,00% |

| statystyka | Statystyka: Mięsień skroniowy lewy(2) x Płeć(2) (Pain drawing statystyka PR-MR .sta) | | |
| --- | --- | --- | --- |
|  | Chi-kwadr. | df | p |
| Chi^2 Pearsona | 2,089664 | df=1 | p=,14830 |
| Chi^2 NW | 2,137030 | df=1 | p=,14378 |
| Chi^2 Yatesa | 1,260895 | df=1 | p=,26148 |
| dokł. Fishera, 1-stronny |  |  | p=,13055 |
| 2-stronny |  |  | p=,20250 |
| Chi^2 McNemara (A/D) | 6,857143 | df=1 | p=,00883 |
| (B/C) | 3,448276 | df=1 | p=,06332 |

| Zwacz prawy | Podsumowująca tabela dwudzielcza: częstości obserwowane (Pain drawing statystyka PR-MR .sta) | | |
| --- | --- | --- | --- |
|  | Płeć  K | Płeć  M | Wiersz  Razem |
| nie | 14 | 1 | 15 |
| % z kolumny | 37,84% | 7,69% |  |
| % z całości | 28,00% | 2,00% | 30,00% |
| tak | 23 | 12 | 35 |
| % z kolumny | 62,16% | 92,31% |  |
| % z całości | 46,00% | 24,00% | 70,00% |
| Razem w kol. | 37 | 13 | 50 |
| % z całości | 74,00% | 26,00% | 100,00% |

| statystyka | Statystyka: Zwacz prawy(2) x Płeć(2) (Pain drawing statystyka PR-MR .sta) | | |
| --- | --- | --- | --- |
|  | Chi-kwadr. | df | p |
| Chi^2 Pearsona | 4,162954 | df=1 | p=,04132 |
| Chi^2 NW | 4,953920 | df=1 | p=,02603 |
| Chi^2 Yatesa | 2,851203 | df=1 | p=,09131 |
| dokł. Fishera, 1-stronny |  |  | p=,03943 |
| 2-stronny |  |  | p=,07555 |
| Chi^2 McNemara (A/D) | ,0384615 | df=1 | p=,84452 |
| (B/C) | 18,37500 | df=1 | p=,00002 |

| zwacz lewy | Podsumowująca tabela dwudzielcza: częstości obserwowane (Pain drawing statystyka PR-MR .sta) | | |
| --- | --- | --- | --- |
|  | Płeć  K | Płeć  M | Wiersz  Razem |
| nie | 11 | 5 | 16 |
| % z kolumny | 29,73% | 38,46% |  |
| % z całości | 22,00% | 10,00% | 32,00% |
| tak | 26 | 8 | 34 |
| % z kolumny | 70,27% | 61,54% |  |
| % z całości | 52,00% | 16,00% | 68,00% |
| Razem w kol. | 37 | 13 | 50 |
| % z całości | 74,00% | 26,00% | 100,00% |

| statystyka | Statystyka: zwacz lewy(2) x Płeć(2) (Pain drawing statystyka PR-MR .sta) | | |
| --- | --- | --- | --- |
|  | Chi-kwadr. | df | p |
| Chi^2 Pearsona | ,3370735 | df=1 | p=,56152 |
| Chi^2 NW | ,3304967 | df=1 | p=,56537 |
| Chi^2 Yatesa | ,0552235 | df=1 | p=,81421 |
| dokł. Fishera, 1-stronny |  |  | p=,39997 |
| 2-stronny |  |  | p=,73099 |
| Chi^2 McNemara (A/D) | ,2105263 | df=1 | p=,64636 |
| (B/C) | 12,90323 | df=1 | p=,00033 |

| TMJ left | Podsumowująca tabela dwudzielcza: częstości obserwowane (Pain drawing statystyka PR-MR .sta) | | |
| --- | --- | --- | --- |
|  | Płeć  K | Płeć  M | Wiersz  Razem |
| nie | 10 | 6 | 16 |
| % z kolumny | 27,03% | 46,15% |  |
| % z całości | 20,00% | 12,00% | 32,00% |
| tak | 27 | 7 | 34 |
| % z kolumny | 72,97% | 53,85% |  |
| % z całości | 54,00% | 14,00% | 68,00% |
| Razem w kol. | 37 | 13 | 50 |
| % z całości | 74,00% | 26,00% | 100,00% |

| statystyka | Statystyka: TMJ left(2) x Płeć(2) (Pain drawing statystyka PR-MR .sta) | | |
| --- | --- | --- | --- |
|  | Chi-kwadr. | df | p |
| Chi^2 Pearsona | 1,617341 | df=1 | p=,20346 |
| Chi^2 NW | 1,561085 | df=1 | p=,21151 |
| Chi^2 Yatesa | ,8577794 | df=1 | p=,35436 |
| dokł. Fishera, 1-stronny |  |  | p=,17649 |
| 2-stronny |  |  | p=,30057 |
| Chi^2 McNemara (A/D) | ,2352941 | df=1 | p=,62763 |
| (B/C) | 12,12121 | df=1 | p=,00050 |

| TMJ Right | Podsumowująca tabela dwudzielcza: częstości obserwowane (Pain drawing statystyka PR-MR .sta) | | |
| --- | --- | --- | --- |
|  | Płeć  K | Płeć  M | Wiersz  Razem |
| nie | 21 | 2 | 23 |
| % z kolumny | 56,76% | 15,38% |  |
| % z całości | 42,00% | 4,00% | 46,00% |
| tak | 16 | 11 | 27 |
| % z kolumny | 43,24% | 84,62% |  |
| % z całości | 32,00% | 22,00% | 54,00% |
| Razem w kol. | 37 | 13 | 50 |
| % z całości | 74,00% | 26,00% | 100,00% |

| statystyka | Statystyka: TMJ Right(2) x Płeć(2) (Pain drawing statystyka PR-MR .sta) | | |
| --- | --- | --- | --- |
|  | Chi-kwadr. | df | p |
| Chi^2 Pearsona | 6,628870 | df=1 | p=,01003 |
| Chi^2 NW | 7,216833 | df=1 | p=,00722 |
| Chi^2 Yatesa | 5,067944 | df=1 | p=,02437 |
| dokł. Fishera, 1-stronny |  |  | p=,01048 |
| 2-stronny |  |  | p=,01192 |
| Chi^2 McNemara (A/D) | 2,531250 | df=1 | p=,11161 |
| (B/C) | 9,388889 | df=1 | p=,00218 |

| pod dolnym katem lopatki lewym | Podsumowująca tabela dwudzielcza: częstości obserwowane (Pain drawing statystyka PR-MR .sta) | | |
| --- | --- | --- | --- |
|  | Płeć  K | Płeć  M | Wiersz  Razem |
| nie | 33 | 13 | 46 |
| % z kolumny | 89,19% | 100,00% |  |
| % z całości | 66,00% | 26,00% | 92,00% |
| tak | 4 | 0 | 4 |
| % z kolumny | 10,81% | 0,00% |  |
| % z całości | 8,00% | 0,00% | 8,00% |
| Razem w kol. | 37 | 13 | 50 |
| % z całości | 74,00% | 26,00% | 100,00% |

| statystyka | Statystyka: pod dolnym katem lopatki lewym(2) x Płeć(2) (Pain drawing statystyka PR-MR .sta) | | |
| --- | --- | --- | --- |
|  | Chi-kwadr. | df | p |
| Chi^2 Pearsona | 1,527615 | df=1 | p=,21647 |
| Chi^2 NW | 2,528866 | df=1 | p=,11178 |
| Chi^2 Yatesa | ,4118458 | df=1 | p=,52103 |
| dokł. Fishera, 1-stronny |  |  | p=,28678 |
| 2-stronny |  |  | p=,56140 |
| Chi^2 McNemara (A/D) | 31,03030 | df=1 | p=,00000 |
| (B/C) | 3,764706 | df=1 | p=,05235 |

| pod katem lopatki prawym | Podsumowująca tabela dwudzielcza: częstości obserwowane (Pain drawing statystyka PR-MR .sta) | | |
| --- | --- | --- | --- |
|  | Płeć  K | Płeć  M | Wiersz  Razem |
| nie | 33 | 13 | 46 |
| % z kolumny | 89,19% | 100,00% |  |
| % z całości | 66,00% | 26,00% | 92,00% |
| tak | 4 | 0 | 4 |
| % z kolumny | 10,81% | 0,00% |  |
| % z całości | 8,00% | 0,00% | 8,00% |
| Razem w kol. | 37 | 13 | 50 |
| % z całości | 74,00% | 26,00% | 100,00% |

| statystyka | Statystyka: pod katem lopatki prawym(2) x Płeć(2) (Pain drawing statystyka PR-MR .sta) | | |
| --- | --- | --- | --- |
|  | Chi-kwadr. | df | p |
| Chi^2 Pearsona | 1,527615 | df=1 | p=,21647 |
| Chi^2 NW | 2,528866 | df=1 | p=,11178 |
| Chi^2 Yatesa | ,4118458 | df=1 | p=,52103 |
| dokł. Fishera, 1-stronny |  |  | p=,28678 |
| 2-stronny |  |  | p=,56140 |
| Chi^2 McNemara (A/D) | 31,03030 | df=1 | p=,00000 |
| (B/C) | 3,764706 | df=1 | p=,05235 |

| MOS prawy | Podsumowująca tabela dwudzielcza: częstości obserwowane (Pain drawing statystyka PR-MR .sta) | | |
| --- | --- | --- | --- |
|  | Płeć  K | Płeć  M | Wiersz  Razem |
| nie | 34 | 10 | 44 |
| % z kolumny | 91,89% | 76,92% |  |
| % z całości | 68,00% | 20,00% | 88,00% |
| tak | 3 | 3 | 6 |
| % z kolumny | 8,11% | 23,08% |  |
| % z całości | 6,00% | 6,00% | 12,00% |
| Razem w kol. | 37 | 13 | 50 |
| % z całości | 74,00% | 26,00% | 100,00% |

| statystyka | Statystyka: MOS prawy(2) x Płeć(2) (Pain drawing statystyka PR-MR .sta) | | |
| --- | --- | --- | --- |
|  | Chi-kwadr. | df | p |
| Chi^2 Pearsona | 2,041202 | df=1 | p=,15309 |
| Chi^2 NW | 1,823455 | df=1 | p=,17690 |
| Chi^2 Yatesa | ,8697946 | df=1 | p=,35101 |
| dokł. Fishera, 1-stronny |  |  | p=,17292 |
| 2-stronny |  |  | p=,17292 |
| Chi^2 McNemara (A/D) | 24,32432 | df=1 | p=,00000 |
| (B/C) | 2,769231 | df=1 | p=,09609 |

| MOS lewy | Podsumowująca tabela dwudzielcza: częstości obserwowane (Pain drawing statystyka PR-MR .sta) | | |
| --- | --- | --- | --- |
|  | Płeć  K | Płeć  M | Wiersz  Razem |
| nie | 34 | 9 | 43 |
| % z kolumny | 91,89% | 69,23% |  |
| % z całości | 68,00% | 18,00% | 86,00% |
| tak | 3 | 4 | 7 |
| % z kolumny | 8,11% | 30,77% |  |
| % z całości | 6,00% | 8,00% | 14,00% |
| Razem w kol. | 37 | 13 | 50 |
| % z całości | 74,00% | 26,00% | 100,00% |

| statystyka | Statystyka: MOS lewy(2) x Płeć(2) (Pain drawing statystyka PR-MR .sta) | | |
| --- | --- | --- | --- |
|  | Chi-kwadr. | df | p |
| Chi^2 Pearsona | 4,103094 | df=1 | p=,04280 |
| Chi^2 NW | 3,624326 | df=1 | p=,05694 |
| Chi^2 Yatesa | 2,436784 | df=1 | p=,11852 |
| dokł. Fishera, 1-stronny |  |  | p=,06485 |
| 2-stronny |  |  | p=,06485 |
| Chi^2 McNemara (A/D) | 22,13158 | df=1 | p=,00000 |
| (B/C) | 2,083333 | df=1 | p=,14891 |

| obrecz barkowa prawa | Podsumowująca tabela dwudzielcza: częstości obserwowane (Pain drawing statystyka PR-MR .sta) | | |
| --- | --- | --- | --- |
|  | Płeć  K | Płeć  M | Wiersz  Razem |
| nie | 24 | 10 | 34 |
| % z kolumny | 64,86% | 76,92% |  |
| % z całości | 48,00% | 20,00% | 68,00% |
| tak | 13 | 3 | 16 |
| % z kolumny | 35,14% | 23,08% |  |
| % z całości | 26,00% | 6,00% | 32,00% |
| Razem w kol. | 37 | 13 | 50 |
| % z całości | 74,00% | 26,00% | 100,00% |

| statystyka | Statystyka: obrecz barkowa prawa(2) x Płeć(2) (Pain drawing statystyka PR-MR .sta) | | |
| --- | --- | --- | --- |
|  | Chi-kwadr. | df | p |
| Chi^2 Pearsona | ,6428091 | df=1 | p=,42270 |
| Chi^2 NW | ,6689796 | df=1 | p=,41341 |
| Chi^2 Yatesa | ,2080913 | df=1 | p=,64827 |
| dokł. Fishera, 1-stronny |  |  | p=,33102 |
| 2-stronny |  |  | p=,50750 |
| Chi^2 McNemara (A/D) | 14,81481 | df=1 | p=,00012 |
| (B/C) | ,1739130 | df=1 | p=,67666 |

| obrecz barkowa lewa | Podsumowująca tabela dwudzielcza: częstości obserwowane (Pain drawing statystyka PR-MR .sta) | | |
| --- | --- | --- | --- |
|  | Płeć  K | Płeć  M | Wiersz  Razem |
| nie | 23 | 11 | 34 |
| % z kolumny | 62,16% | 84,62% |  |
| % z całości | 46,00% | 22,00% | 68,00% |
| tak | 14 | 2 | 16 |
| % z kolumny | 37,84% | 15,38% |  |
| % z całości | 28,00% | 4,00% | 32,00% |
| Razem w kol. | 37 | 13 | 50 |
| % z całości | 74,00% | 26,00% | 100,00% |

| statystyka | Statystyka: obrecz barkowa lewa(2) x Płeć(2) (Pain drawing statystyka PR-MR .sta) | | |
| --- | --- | --- | --- |
|  | Chi-kwadr. | df | p |
| Chi^2 Pearsona | 2,228812 | df=1 | p=,13546 |
| Chi^2 NW | 2,442961 | df=1 | p=,11805 |
| Chi^2 Yatesa | 1,316383 | df=1 | p=,25124 |
| dokł. Fishera, 1-stronny |  |  | p=,12409 |
| 2-stronny |  |  | p=,17917 |
| Chi^2 McNemara (A/D) | 16,00000 | df=1 | p=,00006 |
| (B/C) | ,1600000 | df=1 | p=,68916 |

| gorny otwor klatki piersiowej | Podsumowująca tabela dwudzielcza: częstości obserwowane (Pain drawing statystyka PR-MR .sta) | | |
| --- | --- | --- | --- |
|  | Płeć  K | Płeć  M | Wiersz  Razem |
| nie | 34 | 13 | 47 |
| % z kolumny | 91,89% | 100,00% |  |
| % z całości | 68,00% | 26,00% | 94,00% |
| tak | 3 | 0 | 3 |
| % z kolumny | 8,11% | 0,00% |  |
| % z całości | 6,00% | 0,00% | 6,00% |
| Razem w kol. | 37 | 13 | 50 |
| % z całości | 74,00% | 26,00% | 100,00% |

| statystyka | Statystyka: gorny otwor klatki piersiowej(2) x Płeć(2) (Pain drawing statystyka PR-MR .sta) | | |
| --- | --- | --- | --- |
|  | Chi-kwadr. | df | p |
| Chi^2 Pearsona | 1,121334 | df=1 | p=,28963 |
| Chi^2 NW | 1,873016 | df=1 | p=,17113 |
| Chi^2 Yatesa | ,1444980 | df=1 | p=,70385 |
| dokł. Fishera, 1-stronny |  |  | p=,39643 |
| 2-stronny |  |  | p=,55827 |
| Chi^2 McNemara (A/D) | 32,02941 | df=1 | p=,00000 |
| (B/C) | 5,062500 | df=1 | p=,02445 |

| obrzecz biodrowa prawa | Podsumowująca tabela dwudzielcza: częstości obserwowane (Pain drawing statystyka PR-MR .sta) | | |
| --- | --- | --- | --- |
|  | Płeć  K | Płeć  M | Wiersz  Razem |
| nie | 35 | 13 | 48 |
| % z kolumny | 94,59% | 100,00% |  |
| % z całości | 70,00% | 26,00% | 96,00% |
| tak | 2 | 0 | 2 |
| % z kolumny | 5,41% | 0,00% |  |
| % z całości | 4,00% | 0,00% | 4,00% |
| Razem w kol. | 37 | 13 | 50 |
| % z całości | 74,00% | 26,00% | 100,00% |

| statystyka | Statystyka: obrzecz biodrowa prawa(2) x Płeć(2) (Pain drawing statystyka PR-MR .sta) | | |
| --- | --- | --- | --- |
|  | Chi-kwadr. | df | p |
| Chi^2 Pearsona | ,7319820 | df=1 | p=,39224 |
| Chi^2 NW | 1,233442 | df=1 | p=,26674 |
| Chi^2 Yatesa | ,0010828 | df=1 | p=,97375 |
| dokł. Fishera, 1-stronny |  |  | p=,54367 |
| 2-stronny |  |  | p=1,0000 |
| Chi^2 McNemara (A/D) | 33,02857 | df=1 | p=,00000 |
| (B/C) | 6,666667 | df=1 | p=,00982 |

| obrecz biodrowa lewa | Podsumowująca tabela dwudzielcza: częstości obserwowane (Pain drawing statystyka PR-MR .sta) | | |
| --- | --- | --- | --- |
|  | Płeć  K | Płeć  M | Wiersz  Razem |
| nie | 36 | 13 | 49 |
| % z kolumny | 97,30% | 100,00% |  |
| % z całości | 72,00% | 26,00% | 98,00% |
| tak | 1 | 0 | 1 |
| % z kolumny | 2,70% | 0,00% |  |
| % z całości | 2,00% | 0,00% | 2,00% |
| Razem w kol. | 37 | 13 | 50 |
| % z całości | 74,00% | 26,00% | 100,00% |

| statystyka | Statystyka: obrecz biodrowa lewa(2) x Płeć(2) (Pain drawing statystyka PR-MR .sta) | | |
| --- | --- | --- | --- |
|  | Chi-kwadr. | df | p |
| Chi^2 Pearsona | ,3585218 | df=1 | p=,54933 |
| Chi^2 NW | ,6093494 | df=1 | p=,43503 |
| Chi^2 Yatesa | ,3054860 | df=1 | p=,58046 |
| dokł. Fishera, 1-stronny |  |  | p=,74000 |
| 2-stronny |  |  | p=1,0000 |
| Chi^2 McNemara (A/D) | 34,02778 | df=1 | p=,00000 |
| (B/C) | 8,642858 | df=1 | p=,00328 |

| mostek | Podsumowująca tabela dwudzielcza: częstości obserwowane (Pain drawing statystyka PR-MR .sta) | | |
| --- | --- | --- | --- |
|  | Płeć  K | Płeć  M | Wiersz  Razem |
| nie | 35 | 12 | 47 |
| % z kolumny | 94,59% | 92,31% |  |
| % z całości | 70,00% | 24,00% | 94,00% |
| tak | 2 | 1 | 3 |
| % z kolumny | 5,41% | 7,69% |  |
| % z całości | 4,00% | 2,00% | 6,00% |
| Razem w kol. | 37 | 13 | 50 |
| % z całości | 74,00% | 26,00% | 100,00% |

| statystyka | Statystyka: mostek(2) x Płeć(2) (Pain drawing statystyka PR-MR .sta) | | |
| --- | --- | --- | --- |
|  | Chi-kwadr. | df | p |
| Chi^2 Pearsona | ,0892054 | df=1 | p=,76519 |
| Chi^2 NW | ,0848560 | df=1 | p=,77082 |
| Chi^2 Yatesa | ,1444980 | df=1 | p=,70385 |
| dokł. Fishera, 1-stronny |  |  | p=,60357 |
| 2-stronny |  |  | p=1,0000 |
| Chi^2 McNemara (A/D) | 30,25000 | df=1 | p=,00000 |
| (B/C) | 5,785714 | df=1 | p=,01616 |

| staw kolanowy prawy | Podsumowująca tabela dwudzielcza: częstości obserwowane (Pain drawing statystyka PR-MR .sta) | | |
| --- | --- | --- | --- |
|  | Płeć  K | Płeć  M | Wiersz  Razem |
| nie | 33 | 11 | 44 |
| % z kolumny | 89,19% | 84,62% |  |
| % z całości | 66,00% | 22,00% | 88,00% |
| tak | 4 | 2 | 6 |
| % z kolumny | 10,81% | 15,38% |  |
| % z całości | 8,00% | 4,00% | 12,00% |
| Razem w kol. | 37 | 13 | 50 |
| % z całości | 74,00% | 26,00% | 100,00% |

| statystyka | Statystyka: staw kolanowy prawy(2) x Płeć(2) (Pain drawing statystyka PR-MR .sta) | | |
| --- | --- | --- | --- |
|  | Chi-kwadr. | df | p |
| Chi^2 Pearsona | ,1905752 | df=1 | p=,66244 |
| Chi^2 NW | ,1820290 | df=1 | p=,66964 |
| Chi^2 Yatesa | ,0035438 | df=1 | p=,95253 |
| dokł. Fishera, 1-stronny |  |  | p=,49710 |
| 2-stronny |  |  | p=,64340 |
| Chi^2 McNemara (A/D) | 25,71428 | df=1 | p=,00000 |
| (B/C) | 2,400000 | df=1 | p=,12134 |

| staw kolanowy lewy | Podsumowująca tabela dwudzielcza: częstości obserwowane (Pain drawing statystyka PR-MR .sta) | | |
| --- | --- | --- | --- |
|  | Płeć  K | Płeć  M | Wiersz  Razem |
| nie | 33 | 11 | 44 |
| % z kolumny | 89,19% | 84,62% |  |
| % z całości | 66,00% | 22,00% | 88,00% |
| tak | 4 | 2 | 6 |
| % z kolumny | 10,81% | 15,38% |  |
| % z całości | 8,00% | 4,00% | 12,00% |
| Razem w kol. | 37 | 13 | 50 |
| % z całości | 74,00% | 26,00% | 100,00% |

| statystyka | Statystyka: staw kolanowy lewy(2) x Płeć(2) (Pain drawing statystyka PR-MR .sta) | | |
| --- | --- | --- | --- |
|  | Chi-kwadr. | df | p |
| Chi^2 Pearsona | ,1905752 | df=1 | p=,66244 |
| Chi^2 NW | ,1820290 | df=1 | p=,66964 |
| Chi^2 Yatesa | ,0035438 | df=1 | p=,95253 |
| dokł. Fishera, 1-stronny |  |  | p=,49710 |
| 2-stronny |  |  | p=,64340 |
| Chi^2 McNemara (A/D) | 25,71428 | df=1 | p=,00000 |
| (B/C) | 2,400000 | df=1 | p=,12134 |

| podudzie prawe | Podsumowująca tabela dwudzielcza: częstości obserwowane (Pain drawing statystyka PR-MR .sta) | | |
| --- | --- | --- | --- |
|  | Płeć  K | Płeć  M | Wiersz  Razem |
| nie | 35 | 11 | 46 |
| % z kolumny | 94,59% | 84,62% |  |
| % z całości | 70,00% | 22,00% | 92,00% |
| tak | 2 | 2 | 4 |
| % z kolumny | 5,41% | 15,38% |  |
| % z całości | 4,00% | 4,00% | 8,00% |
| Razem w kol. | 37 | 13 | 50 |
| % z całości | 74,00% | 26,00% | 100,00% |

| statystyka | Statystyka: podudzie prawe(2) x Płeć(2) (Pain drawing statystyka PR-MR .sta) | | |
| --- | --- | --- | --- |
|  | Chi-kwadr. | df | p |
| Chi^2 Pearsona | 1,301636 | df=1 | p=,25391 |
| Chi^2 NW | 1,153566 | df=1 | p=,28280 |
| Chi^2 Yatesa | ,2988566 | df=1 | p=,58460 |
| dokł. Fishera, 1-stronny |  |  | p=,27462 |
| 2-stronny |  |  | p=,27462 |
| Chi^2 McNemara (A/D) | 27,67568 | df=1 | p=,00000 |
| (B/C) | 4,923077 | df=1 | p=,02650 |

| podudzie lewe | Podsumowująca tabela dwudzielcza: częstości obserwowane (Pain drawing statystyka PR-MR .sta) | | |
| --- | --- | --- | --- |
|  | Płeć  K | Płeć  M | Wiersz  Razem |
| nie | 35 | 11 | 46 |
| % z kolumny | 97,22% | 84,62% |  |
| % z całości | 71,43% | 22,45% | 93,88% |
| tak | 1 | 2 | 3 |
| % z kolumny | 2,78% | 15,38% |  |
| % z całości | 2,04% | 4,08% | 6,12% |
| Razem w kol. | 36 | 13 | 49 |
| % z całości | 73,47% | 26,53% | 100,00% |

| statystyka | Statystyka: podudzie lewe(2) x Płeć(2) (Pain drawing statystyka PR-MR .sta) | | |
| --- | --- | --- | --- |
|  | Chi-kwadr. | df | p |
| Chi^2 Pearsona | 2,641041 | df=1 | p=,10414 |
| Chi^2 NW | 2,270309 | df=1 | p=,13187 |
| Chi^2 Yatesa | ,9030449 | df=1 | p=,34197 |
| dokł. Fishera, 1-stronny |  |  | p=,16793 |
| 2-stronny |  |  | p=,16793 |
| Chi^2 McNemara (A/D) | 27,67568 | df=1 | p=,00000 |
| (B/C) | 6,750000 | df=1 | p=,00937 |

| zeby 18-14 | Podsumowująca tabela dwudzielcza: częstości obserwowane (Pain drawing statystyka PR-MR .sta) | | |
| --- | --- | --- | --- |
|  | Płeć  K | Płeć  M | Wiersz  Razem |
| nie | 32 | 12 | 44 |
| % z kolumny | 86,49% | 92,31% |  |
| % z całości | 64,00% | 24,00% | 88,00% |
| tak | 5 | 1 | 6 |
| % z kolumny | 13,51% | 7,69% |  |
| % z całości | 10,00% | 2,00% | 12,00% |
| Razem w kol. | 37 | 13 | 50 |
| % z całości | 74,00% | 26,00% | 100,00% |

| statystyka | Statystyka: zeby 18-14(2) x Płeć(2) (Pain drawing statystyka PR-MR .sta) | | |
| --- | --- | --- | --- |
|  | Chi-kwadr. | df | p |
| Chi^2 Pearsona | ,3087003 | df=1 | p=,57848 |
| Chi^2 NW | ,3351268 | df=1 | p=,56266 |
| Chi^2 Yatesa | ,0035438 | df=1 | p=,95253 |
| dokł. Fishera, 1-stronny |  |  | p=,50290 |
| 2-stronny |  |  | p=1,0000 |
| Chi^2 McNemara (A/D) | 27,27273 | df=1 | p=,00000 |
| (B/C) | 2,117647 | df=1 | p=,14561 |

| zeby 13-11 | Podsumowująca tabela dwudzielcza: częstości obserwowane (Pain drawing statystyka PR-MR .sta) | | |
| --- | --- | --- | --- |
|  | Płeć  K | Płeć  M | Wiersz  Razem |
| nie | 36 | 12 | 48 |
| % z kolumny | 97,30% | 92,31% |  |
| % z całości | 72,00% | 24,00% | 96,00% |
| tak | 1 | 1 | 2 |
| % z kolumny | 2,70% | 7,69% |  |
| % z całości | 2,00% | 2,00% | 4,00% |
| Razem w kol. | 37 | 13 | 50 |
| % z całości | 74,00% | 26,00% | 100,00% |

| statystyka | Statystyka: zeby 13-11(2) x Płeć(2) (Pain drawing statystyka PR-MR .sta) | | |
| --- | --- | --- | --- |
|  | Chi-kwadr. | df | p |
| Chi^2 Pearsona | ,6237006 | df=1 | p=,42968 |
| Chi^2 NW | ,5489291 | df=1 | p=,45876 |
| Chi^2 Yatesa | ,0010828 | df=1 | p=,97375 |
| dokł. Fishera, 1-stronny |  |  | p=,45633 |
| 2-stronny |  |  | p=,45633 |
| Chi^2 McNemara (A/D) | 31,24324 | df=1 | p=,00000 |
| (B/C) | 7,692307 | df=1 | p=,00555 |

| zeby 21-23 | Podsumowująca tabela dwudzielcza: częstości obserwowane (Pain drawing statystyka PR-MR .sta) | | |
| --- | --- | --- | --- |
|  | Płeć  K | Płeć  M | Wiersz  Razem |
| nie | 36 | 12 | 48 |
| % z kolumny | 97,30% | 92,31% |  |
| % z całości | 72,00% | 24,00% | 96,00% |
| tak | 1 | 1 | 2 |
| % z kolumny | 2,70% | 7,69% |  |
| % z całości | 2,00% | 2,00% | 4,00% |
| Razem w kol. | 37 | 13 | 50 |
| % z całości | 74,00% | 26,00% | 100,00% |

| statystyka | Statystyka: zeby 21-23(2) x Płeć(2) (Pain drawing statystyka PR-MR .sta) | | |
| --- | --- | --- | --- |
|  | Chi-kwadr. | df | p |
| Chi^2 Pearsona | ,6237006 | df=1 | p=,42968 |
| Chi^2 NW | ,5489291 | df=1 | p=,45876 |
| Chi^2 Yatesa | ,0010828 | df=1 | p=,97375 |
| dokł. Fishera, 1-stronny |  |  | p=,45633 |
| 2-stronny |  |  | p=,45633 |
| Chi^2 McNemara (A/D) | 31,24324 | df=1 | p=,00000 |
| (B/C) | 7,692307 | df=1 | p=,00555 |

| zeby 24-28 | Podsumowująca tabela dwudzielcza: częstości obserwowane (Pain drawing statystyka PR-MR .sta) | | |
| --- | --- | --- | --- |
|  | Płeć  K | Płeć  M | Wiersz  Razem |
| nie | 32 | 12 | 44 |
| % z kolumny | 86,49% | 92,31% |  |
| % z całości | 64,00% | 24,00% | 88,00% |
| tak | 5 | 1 | 6 |
| % z kolumny | 13,51% | 7,69% |  |
| % z całości | 10,00% | 2,00% | 12,00% |
| Razem w kol. | 37 | 13 | 50 |
| % z całości | 74,00% | 26,00% | 100,00% |

| statystyka | Statystyka: zeby 24-28(2) x Płeć(2) (Pain drawing statystyka PR-MR .sta) | | |
| --- | --- | --- | --- |
|  | Chi-kwadr. | df | p |
| Chi^2 Pearsona | ,3087003 | df=1 | p=,57848 |
| Chi^2 NW | ,3351268 | df=1 | p=,56266 |
| Chi^2 Yatesa | ,0035438 | df=1 | p=,95253 |
| dokł. Fishera, 1-stronny |  |  | p=,50290 |
| 2-stronny |  |  | p=1,0000 |
| Chi^2 McNemara (A/D) | 27,27273 | df=1 | p=,00000 |
| (B/C) | 2,117647 | df=1 | p=,14561 |

| zeby 34-38 | Podsumowująca tabela dwudzielcza: częstości obserwowane (Pain drawing statystyka PR-MR .sta) | | |
| --- | --- | --- | --- |
|  | Płeć  K | Płeć  M | Wiersz  Razem |
| nie | 33 | 11 | 44 |
| % z kolumny | 89,19% | 84,62% |  |
| % z całości | 66,00% | 22,00% | 88,00% |
| tak | 4 | 2 | 6 |
| % z kolumny | 10,81% | 15,38% |  |
| % z całości | 8,00% | 4,00% | 12,00% |
| Razem w kol. | 37 | 13 | 50 |
| % z całości | 74,00% | 26,00% | 100,00% |

| statystyka | Statystyka: zeby 34-38(2) x Płeć(2) (Pain drawing statystyka PR-MR .sta) | | |
| --- | --- | --- | --- |
|  | Chi-kwadr. | df | p |
| Chi^2 Pearsona | ,1905752 | df=1 | p=,66244 |
| Chi^2 NW | ,1820290 | df=1 | p=,66964 |
| Chi^2 Yatesa | ,0035438 | df=1 | p=,95253 |
| dokł. Fishera, 1-stronny |  |  | p=,49710 |
| 2-stronny |  |  | p=,64340 |
| Chi^2 McNemara (A/D) | 25,71428 | df=1 | p=,00000 |
| (B/C) | 2,400000 | df=1 | p=,12134 |

| zeby 31-33 | Podsumowująca tabela dwudzielcza: częstości obserwowane (Pain drawing statystyka PR-MR .sta) | | |
| --- | --- | --- | --- |
|  | Płeć  K | Płeć  M | Wiersz  Razem |
| nie | 35 | 12 | 47 |
| % z kolumny | 94,59% | 92,31% |  |
| % z całości | 70,00% | 24,00% | 94,00% |
| tak | 2 | 1 | 3 |
| % z kolumny | 5,41% | 7,69% |  |
| % z całości | 4,00% | 2,00% | 6,00% |
| Razem w kol. | 37 | 13 | 50 |
| % z całości | 74,00% | 26,00% | 100,00% |

| statystyka | Statystyka: zeby 31-33(2) x Płeć(2) (Pain drawing statystyka PR-MR .sta) | | |
| --- | --- | --- | --- |
|  | Chi-kwadr. | df | p |
| Chi^2 Pearsona | ,0892054 | df=1 | p=,76519 |
| Chi^2 NW | ,0848560 | df=1 | p=,77082 |
| Chi^2 Yatesa | ,1444980 | df=1 | p=,70385 |
| dokł. Fishera, 1-stronny |  |  | p=,60357 |
| 2-stronny |  |  | p=1,0000 |
| Chi^2 McNemara (A/D) | 30,25000 | df=1 | p=,00000 |
| (B/C) | 5,785714 | df=1 | p=,01616 |

| zeby 41-43 | Podsumowująca tabela dwudzielcza: częstości obserwowane (Pain drawing statystyka PR-MR .sta) | | |
| --- | --- | --- | --- |
|  | Płeć  K | Płeć  M | Wiersz  Razem |
| nie | 34 | 12 | 46 |
| % z kolumny | 91,89% | 92,31% |  |
| % z całości | 68,00% | 24,00% | 92,00% |
| tak | 3 | 1 | 4 |
| % z kolumny | 8,11% | 7,69% |  |
| % z całości | 6,00% | 2,00% | 8,00% |
| Razem w kol. | 37 | 13 | 50 |
| % z całości | 74,00% | 26,00% | 100,00% |

| statystyka | Statystyka: zeby 41-43(2) x Płeć(2) (Pain drawing statystyka PR-MR .sta) | | |
| --- | --- | --- | --- |
|  | Chi-kwadr. | df | p |
| Chi^2 Pearsona | ,0022598 | df=1 | p=,96209 |
| Chi^2 NW | ,0022773 | df=1 | p=,96194 |
| Chi^2 Yatesa | ,2988566 | df=1 | p=,58460 |
| dokł. Fishera, 1-stronny |  |  | p=,72538 |
| 2-stronny |  |  | p=1,0000 |
| Chi^2 McNemara (A/D) | 29,25714 | df=1 | p=,00000 |
| (B/C) | 4,266667 | df=1 | p=,03887 |

| zeby 44-48 | Podsumowująca tabela dwudzielcza: częstości obserwowane (Pain drawing statystyka PR-MR .sta) | | |
| --- | --- | --- | --- |
|  | Płeć  K | Płeć  M | Wiersz  Razem |
| nie | 32 | 11 | 43 |
| % z kolumny | 86,49% | 84,62% |  |
| % z całości | 64,00% | 22,00% | 86,00% |
| tak | 5 | 2 | 7 |
| % z kolumny | 13,51% | 15,38% |  |
| % z całości | 10,00% | 4,00% | 14,00% |
| Razem w kol. | 37 | 13 | 50 |
| % z całości | 74,00% | 26,00% | 100,00% |

| statystyka | Statystyka: zeby 44-48(2) x Płeć(2) (Pain drawing statystyka PR-MR .sta) | | |
| --- | --- | --- | --- |
|  | Chi-kwadr. | df | p |
| Chi^2 Pearsona | ,0279733 | df=1 | p=,86717 |
| Chi^2 NW | ,0275013 | df=1 | p=,86829 |
| Chi^2 Yatesa | ,0884094 | df=1 | p=,76621 |
| dokł. Fishera, 1-stronny |  |  | p=,59435 |
| 2-stronny |  |  | p=1,0000 |
| Chi^2 McNemara (A/D) | 24,73529 | df=1 | p=,00000 |
| (B/C) | 1,562500 | df=1 | p=,21130 |

| podniebienie | Podsumowująca tabela dwudzielcza: częstości obserwowane (Pain drawing statystyka PR-MR .sta) | | |
| --- | --- | --- | --- |
|  | Płeć  K | Płeć  M | Wiersz  Razem |
| nie | 36 | 13 | 49 |
| % z kolumny | 97,30% | 100,00% |  |
| % z całości | 72,00% | 26,00% | 98,00% |
| tak | 1 | 0 | 1 |
| % z kolumny | 2,70% | 0,00% |  |
| % z całości | 2,00% | 0,00% | 2,00% |
| Razem w kol. | 37 | 13 | 50 |
| % z całości | 74,00% | 26,00% | 100,00% |

| statystyka | Statystyka: podniebienie(2) x Płeć(2) (Pain drawing statystyka PR-MR .sta) | | |
| --- | --- | --- | --- |
|  | Chi-kwadr. | df | p |
| Chi^2 Pearsona | ,3585218 | df=1 | p=,54933 |
| Chi^2 NW | ,6093494 | df=1 | p=,43503 |
| Chi^2 Yatesa | ,3054860 | df=1 | p=,58046 |
| dokł. Fishera, 1-stronny |  |  | p=,74000 |
| 2-stronny |  |  | p=1,0000 |
| Chi^2 McNemara (A/D) | 34,02778 | df=1 | p=,00000 |
| (B/C) | 8,642858 | df=1 | p=,00328 |

| dlon prawa | Podsumowująca tabela dwudzielcza: częstości obserwowane (Pain drawing statystyka PR-MR .sta) | | |
| --- | --- | --- | --- |
|  | Płeć  K | Płeć  M | Wiersz  Razem |
| nie | 36 | 13 | 49 |
| % z kolumny | 97,30% | 100,00% |  |
| % z całości | 72,00% | 26,00% | 98,00% |
| tak | 1 | 0 | 1 |
| % z kolumny | 2,70% | 0,00% |  |
| % z całości | 2,00% | 0,00% | 2,00% |
| Razem w kol. | 37 | 13 | 50 |
| % z całości | 74,00% | 26,00% | 100,00% |

| statystyka | Statystyka: dlon prawa(2) x Płeć(2) (Pain drawing statystyka PR-MR .sta) | | |
| --- | --- | --- | --- |
|  | Chi-kwadr. | df | p |
| Chi^2 Pearsona | ,3585218 | df=1 | p=,54933 |
| Chi^2 NW | ,6093494 | df=1 | p=,43503 |
| Chi^2 Yatesa | ,3054860 | df=1 | p=,58046 |
| dokł. Fishera, 1-stronny |  |  | p=,74000 |
| 2-stronny |  |  | p=1,0000 |
| Chi^2 McNemara (A/D) | 34,02778 | df=1 | p=,00000 |
| (B/C) | 8,642858 | df=1 | p=,00328 |

| dlon lewa | Podsumowująca tabela dwudzielcza: częstości obserwowane (Pain drawing statystyka PR-MR .sta) | | |
| --- | --- | --- | --- |
|  | Płeć  K | Płeć  M | Wiersz  Razem |
| nie | 35 | 13 | 48 |
| % z kolumny | 94,59% | 100,00% |  |
| % z całości | 70,00% | 26,00% | 96,00% |
| tak | 2 | 0 | 2 |
| % z kolumny | 5,41% | 0,00% |  |
| % z całości | 4,00% | 0,00% | 4,00% |
| Razem w kol. | 37 | 13 | 50 |
| % z całości | 74,00% | 26,00% | 100,00% |

| statystyka | Statystyka: dlon lewa(2) x Płeć(2) (Pain drawing statystyka PR-MR .sta) | | |
| --- | --- | --- | --- |
|  | Chi-kwadr. | df | p |
| Chi^2 Pearsona | ,7319820 | df=1 | p=,39224 |
| Chi^2 NW | 1,233442 | df=1 | p=,26674 |
| Chi^2 Yatesa | ,0010828 | df=1 | p=,97375 |
| dokł. Fishera, 1-stronny |  |  | p=,54367 |
| 2-stronny |  |  | p=1,0000 |
| Chi^2 McNemara (A/D) | 33,02857 | df=1 | p=,00000 |
| (B/C) | 6,666667 | df=1 | p=,00982 |

| wiezadlo skrzydlowo-żuchwowe lewe | Podsumowująca tabela dwudzielcza: częstości obserwowane (Pain drawing statystyka PR-MR .sta) | | |
| --- | --- | --- | --- |
|  | Płeć  K | Płeć  M | Wiersz  Razem |
| nie | 27 | 12 | 39 |
| % z kolumny | 72,97% | 92,31% |  |
| % z całości | 54,00% | 24,00% | 78,00% |
| tak | 10 | 1 | 11 |
| % z kolumny | 27,03% | 7,69% |  |
| % z całości | 20,00% | 2,00% | 22,00% |
| Razem w kol. | 37 | 13 | 50 |
| % z całości | 74,00% | 26,00% | 100,00% |

| statystyka | Statystyka: wiezadlo skrzydlowo-żuchwowe lewe(2) x Płeć(2) (Pain drawing statystyka PR-MR .sta) | | |
| --- | --- | --- | --- |
|  | Chi-kwadr. | df | p |
| Chi^2 Pearsona | 2,095721 | df=1 | p=,14771 |
| Chi^2 NW | 2,458839 | df=1 | p=,11687 |
| Chi^2 Yatesa | 1,120432 | df=1 | p=,28983 |
| dokł. Fishera, 1-stronny |  |  | p=,14412 |
| 2-stronny |  |  | p=,24755 |
| Chi^2 McNemara (A/D) | 22,32143 | df=1 | p=,00000 |
| (B/C) | ,0454545 | df=1 | p=,83117 |

| wiezadlo skrzydlowo-żuchwowe prawe | Podsumowująca tabela dwudzielcza: częstości obserwowane (Pain drawing statystyka PR-MR .sta) | | |
| --- | --- | --- | --- |
|  | Płeć  K | Płeć  M | Wiersz  Razem |
| nie | 31 | 11 | 42 |
| % z kolumny | 83,78% | 84,62% |  |
| % z całości | 62,00% | 22,00% | 84,00% |
| tak | 6 | 2 | 8 |
| % z kolumny | 16,22% | 15,38% |  |
| % z całości | 12,00% | 4,00% | 16,00% |
| Razem w kol. | 37 | 13 | 50 |
| % z całości | 74,00% | 26,00% | 100,00% |

| statystyka | Statystyka: wiezadlo skrzydlowo-żuchwowe prawe(2) x Płeć(2) (Pain drawing statystyka PR-MR .sta) | | |
| --- | --- | --- | --- |
|  | Chi-kwadr. | df | p |
| Chi^2 Pearsona | ,0049500 | df=1 | p=,94391 |
| Chi^2 NW | ,0049841 | df=1 | p=,94372 |
| Chi^2 Yatesa | ,1364345 | df=1 | p=,71185 |
| dokł. Fishera, 1-stronny |  |  | p=,65896 |
| 2-stronny |  |  | p=1,0000 |
| Chi^2 McNemara (A/D) | 23,75758 | df=1 | p=,00000 |
| (B/C) | ,9411765 | df=1 | p=,33198 |

| oko prawe oko lewe | Podsumowująca tabela dwudzielcza: częstości obserwowane (Pain drawing statystyka PR-MR .sta) | | |
| --- | --- | --- | --- |
|  | Płeć  K | Płeć  M | Wiersz  Razem |
| nie | 36 | 13 | 49 |
| % z kolumny | 97,30% | 100,00% |  |
| % z całości | 72,00% | 26,00% | 98,00% |
| tak | 1 | 0 | 1 |
| % z kolumny | 2,70% | 0,00% |  |
| % z całości | 2,00% | 0,00% | 2,00% |
| Razem w kol. | 37 | 13 | 50 |
| % z całości | 74,00% | 26,00% | 100,00% |

| statystyka | Statystyka: oko prawe oko lewe(2) x Płeć(2) (Pain drawing statystyka PR-MR .sta) | | |
| --- | --- | --- | --- |
|  | Chi-kwadr. | df | p |
| Chi^2 Pearsona | ,3585218 | df=1 | p=,54933 |
| Chi^2 NW | ,6093494 | df=1 | p=,43503 |
| Chi^2 Yatesa | ,3054860 | df=1 | p=,58046 |
| dokł. Fishera, 1-stronny |  |  | p=,74000 |
| 2-stronny |  |  | p=1,0000 |
| Chi^2 McNemara (A/D) | 34,02778 | df=1 | p=,00000 |
| (B/C) | 8,642858 | df=1 | p=,00328 |

| TMD Pain Screener Level | Podsumowująca tabela dwudzielcza: częstości obserwowane (Arkusz1 PR.sta) | | |
| --- | --- | --- | --- |
|  | Płeć  K | Płeć  M | Wiersz  Razem |
| 0 | 11 | 4 | 15 |
| % z kolumny | 29,73% | 30,77% |  |
| % z całości | 22,00% | 8,00% | 30,00% |
| 1 | 26 | 9 | 35 |
| % z kolumny | 70,27% | 69,23% |  |
| % z całości | 52,00% | 18,00% | 70,00% |
| Razem w kol. | 37 | 13 | 50 |
| % z całości | 74,00% | 26,00% | 100,00% |

| statystyka | Statystyka: TMD Pain Screener Level(2) x Płeć(2) (Arkusz1 PR.sta) | | |
| --- | --- | --- | --- |
|  | Chi-kwadr. | df | p |
| Chi^2 Pearsona | ,0049500 | df=1 | p=,94391 |
| Chi^2 NW | ,0049346 | df=1 | p=,94400 |
| Chi^2 Yatesa | ,0792001 | df=1 | p=,77838 |
| dokł. Fishera, 1-stronny |  |  | p=,60173 |
| 2-stronny |  |  | p=1,0000 |
| Chi^2 McNemara (A/D) | ,0500000 | df=1 | p=,82306 |
| (B/C) | 14,70000 | df=1 | p=,00013 |

| GCPS Grade | Podsumowująca tabela dwudzielcza: częstości obserwowane (Arkusz1 PR.sta) | | |
| --- | --- | --- | --- |
|  | Płeć  K | Płeć  M | Wiersz  Razem |
| 0 | 2 | 1 | 3 |
| % z kolumny | 5,41% | 7,69% |  |
| % z całości | 4,00% | 2,00% | 6,00% |
| 1 | 24 | 6 | 30 |
| % z kolumny | 64,86% | 46,15% |  |
| % z całości | 48,00% | 12,00% | 60,00% |
| 2 | 4 | 2 | 6 |
| % z kolumny | 10,81% | 15,38% |  |
| % z całości | 8,00% | 4,00% | 12,00% |
| 3 | 2 | 4 | 6 |
| % z kolumny | 5,41% | 30,77% |  |
| % z całości | 4,00% | 8,00% | 12,00% |
| 4 | 5 | 0 | 5 |
| % z kolumny | 13,51% | 0,00% |  |
| % z całości | 10,00% | 0,00% | 10,00% |
| Razem w kol. | 37 | 13 | 50 |
| % z całości | 74,00% | 26,00% | 100,00% |

| statystyka | Statystyka: GCPS Grade(5) x Płeć(2) (Arkusz1 PR.sta) | | |
| --- | --- | --- | --- |
|  | Chi-kwadr. | df | p |
| Chi^2 Pearsona | 7,726958 | df=4 | p=,10211 |
| Chi^2 NW | 8,186121 | df=4 | p=,08499 |

| TMD-PSc kod | Podsumowująca tabela dwudzielcza: częstości obserwowane (Arkusz1 PR.sta) | | |
| --- | --- | --- | --- |
|  | Płeć  K | Płeć  M | Wiersz  Razem |
| 0-3 | 11 | 4 | 15 |
| % z kolumny | 29,73% | 30,77% |  |
| % z całości | 22,00% | 8,00% | 30,00% |
| 4-6 | 26 | 9 | 35 |
| % z kolumny | 70,27% | 69,23% |  |
| % z całości | 52,00% | 18,00% | 70,00% |
| Razem w kol. | 37 | 13 | 50 |
| % z całości | 74,00% | 26,00% | 100,00% |

| statystyka | Statystyka: TMD-PSc kod(2) x Płeć(2) (Arkusz1 PR.sta) | | |
| --- | --- | --- | --- |
|  | Chi-kwadr. | df | p |
| Chi^2 Pearsona | ,0049500 | df=1 | p=,94391 |
| Chi^2 NW | ,0049346 | df=1 | p=,94400 |
| Chi^2 Yatesa | ,0792001 | df=1 | p=,77838 |
| dokł. Fishera, 1-stronny |  |  | p=,60173 |
| 2-stronny |  |  | p=1,0000 |
| Chi^2 McNemara (A/D) | ,0500000 | df=1 | p=,82306 |
| (B/C) | 14,70000 | df=1 | p=,00013 |

| Zmienna | Statystyki opisowe (Arkusz1 PR.sta) | | | | | | | | |
| --- | --- | --- | --- | --- | --- | --- | --- | --- | --- |
|  | Nważnych | Średnia | Mediana | Minimum | Maksimum | Dolny  Kwartyl. | Górny  Kwartyl. | Odch.std | Skośność |
| TMD Pain Screener punkty | 50 | 4,340000 | 4,000000 | 0,00 | 7,000000 | 3,000000 | 6,000000 | 1,546688 | -0,635043 |

| Zmienna | Statystyki opisowe (Arkusz1 PR.sta)Warunek uwzględniania: v2=0 | | | | | | | | |
| --- | --- | --- | --- | --- | --- | --- | --- | --- | --- |
|  | Nważnych | Średnia | Mediana | Minimum | Maksimum | Dolny  Kwartyl. | Górny  Kwartyl. | Odch.std | Skośność |
| TMD Pain Screener punkty | 37 | 4,270270 | 4,000000 | 1,000000 | 6,000000 | 3,000000 | 6,000000 | 1,426897 | -0,446364 |

| Zmienna | Statystyki opisowe (Arkusz1 PR.sta)Warunek uwzględniania: v2=1 | | | | | | | | |
| --- | --- | --- | --- | --- | --- | --- | --- | --- | --- |
|  | Nważnych | Średnia | Mediana | Minimum | Maksimum | Dolny  Kwartyl. | Górny  Kwartyl. | Odch.std | Skośność |
| TMD Pain Screener punkty | 13 | 4,538462 | 5,000000 | 0,00 | 7,000000 | 3,000000 | 6,000000 | 1,898042 | -1,10707 |

| Zmienna | Statystyki opisowe (Arkusz1 PR.sta)Warunek uwzględniania: v7=0 | | | | | | | | |
| --- | --- | --- | --- | --- | --- | --- | --- | --- | --- |
|  | Nważnych | Średnia | Mediana | Minimum | Maksimum | Dolny  Kwartyl. | Górny  Kwartyl. | Odch.std | Skośność |
| TMD Pain Screener punkty | 15 | 2,466667 | 3,000000 | 0,00 | 3,000000 | 2,000000 | 3,000000 | 0,990430 | -1,67548 |

| Zmienna | Statystyki opisowe (Arkusz1 PR.sta)Warunek uwzględniania: v7=1 | | | | | | | | |
| --- | --- | --- | --- | --- | --- | --- | --- | --- | --- |
|  | Nważnych | Średnia | Mediana | Minimum | Maksimum | Dolny  Kwartyl. | Górny  Kwartyl. | Odch.std | Skośność |
| TMD Pain Screener punkty | 35 | 5,142857 | 5,000000 | 4,000000 | 7,000000 | 4,000000 | 6,000000 | 0,912103 | -0,050338 |

| Zmienna | Statystyki opisowe (Arkusz1 PR.sta)Warunek uwzględniania: v7=0 and v2=0 | | | | | | | | |
| --- | --- | --- | --- | --- | --- | --- | --- | --- | --- |
|  | Nważnych | Średnia | Mediana | Minimum | Maksimum | Dolny  Kwartyl. | Górny  Kwartyl. | Odch.std | Skośność |
| TMD Pain Screener punkty | 11 | 2,545455 | 3,000000 | 1,000000 | 3,000000 | 2,000000 | 3,000000 | 0,820200 | -1,50480 |

| Zmienna | Statystyki opisowe (Arkusz1 PR.sta)Warunek uwzględniania: v7=0 and v2=1 | | | | | | | | |
| --- | --- | --- | --- | --- | --- | --- | --- | --- | --- |
|  | Nważnych | Średnia | Mediana | Minimum | Maksimum | Dolny  Kwartyl. | Górny  Kwartyl. | Odch.std | Skośność |
| TMD Pain Screener punkty | 4 | 2,250000 | 3,000000 | 0,00 | 3,000000 | 1,500000 | 3,000000 | 1,500000 | -2,00000 |

| Zmienna | Statystyki opisowe (Arkusz1 PR.sta)Warunek uwzględniania: v7=1 and v2=0 | | | | | | | | |
| --- | --- | --- | --- | --- | --- | --- | --- | --- | --- |
|  | Nważnych | Średnia | Mediana | Minimum | Maksimum | Dolny  Kwartyl. | Górny  Kwartyl. | Odch.std | Skośność |
| TMD Pain Screener punkty | 26 | 5,000000 | 5,000000 | 4,000000 | 6,000000 | 4,000000 | 6,000000 | 0,894427 | 0,000000 |

| Zmienna | Statystyki opisowe (Arkusz1 PR.sta)Warunek uwzględniania: v7=1 and v2=1 | | | | | | | | |
| --- | --- | --- | --- | --- | --- | --- | --- | --- | --- |
|  | Nważnych | Średnia | Mediana | Minimum | Maksimum | Dolny  Kwartyl. | Górny  Kwartyl. | Odch.std | Skośność |
| TMD Pain Screener punkty | 9 | 5,555556 | 6,000000 | 4,000000 | 7,000000 | 5,000000 | 6,000000 | 0,881917 | -0,214051 |

| Zmienna | Testy t; Grupująca: Płeć (Arkusz1 PR.sta)  Grupa 1: K  Grupa 2 M | | | | | | | | | |
| --- | --- | --- | --- | --- | --- | --- | --- | --- | --- | --- |
|  | Średnia  K | Średnia  M | t | df | p | Nważnych  K | Nważnych  M | Odch.std  K | Odch.std  M | iloraz F  Wariancje |
| TMD Pain Screener punkty | 4,270270 | 4,538462 | -0,533872 | 48 | 0,595894 | 37 | 13 | 1,426897 | 1,898042 | 1,769401 |

| Zmienna | Testy t; Grupująca: Płeć (Arkusz1 PR.sta)  Grupa 1: K  Grupa 2 M |
| --- | --- |
|  | p  Wariancje |
| TMD Pain Screener punkty | 0,184307 |

| Zmienna | Testy t; Grupująca: Płeć (Arkusz1 PR.sta)  Grupa 1: K  Grupa 2 M  Warunek uwzględniania: v7=1 | | | | | | | | | |
| --- | --- | --- | --- | --- | --- | --- | --- | --- | --- | --- |
|  | Średnia  K | Średnia  M | t | df | p | Nważnych  K | Nważnych  M | Odch.std  K | Odch.std  M | iloraz F  Wariancje |
| TMD Pain Screener punkty | 5,000000 | 5,555556 | -1,61148 | 33 | 0,116601 | 26 | 9 | 0,894427 | 0,881917 | 1,028571 |

| Zmienna | Testy t; Grupująca: Płeć (Arkusz1 PR.sta)  Grupa 1: K  Grupa 2 M  Warunek uwzględniania: v7=1 |
| --- | --- |
|  | p  Wariancje |
| TMD Pain Screener punkty | 1,000000 |

| Zmienna | Test U Manna-Whitneya (z poprawką na ciągłość) (Arkusz1 PR.sta)  Względem zmiennej: Płeć  Zaznaczone wyniki są istotne z p <,05000 | | | | | | | | | |
| --- | --- | --- | --- | --- | --- | --- | --- | --- | --- | --- |
|  | Sum.rang  K | Sum.rang  M | U | Z | p | Z  popraw. | p | N ważn.  K | N ważn.  M | 2*1str.  dokł. p |
| TMD Pain Screener punkty | 910,0000 | 365,0000 | 207,0000 | -0,729872 | 0,465469 | -0,748356 | 0,454246 | 37 | 13 | 0,470185 |

| Zmienna | Test U Manna-Whitneya (z poprawką na ciągłość) (Arkusz1 PR.sta)  Względem zmiennej: Płeć  Zaznaczone wyniki są istotne z p <,05000  Warunek uwzględniania: v7=0 | | | | | | | | | |
| --- | --- | --- | --- | --- | --- | --- | --- | --- | --- | --- |
|  | Sum.rang  K | Sum.rang  M | U | Z | p | Z  popraw. | p | N ważn.  K | N ważn.  M | 2*1str.  dokł. p |
| TMD Pain Screener punkty | 89,00000 | 31,00000 | 21,00000 | 0,065279 | 0,947952 | 0,083901 | 0,933135 | 11 | 4 | 0,949451 |

| Zmienna | Test U Manna-Whitneya (z poprawką na ciągłość) (Arkusz1 PR.sta)  Względem zmiennej: Płeć  Zaznaczone wyniki są istotne z p <,05000  Warunek uwzględniania: v7=1 | | | | | | | | | |
| --- | --- | --- | --- | --- | --- | --- | --- | --- | --- | --- |
|  | Sum.rang  K | Sum.rang  M | U | Z | p | Z  popraw. | p | N ważn.  K | N ważn.  M | 2*1str.  dokł. p |
| TMD Pain Screener punkty | 431,0000 | 199,0000 | 80,00000 | -1,37760 | 0,168327 | -1,46136 | 0,143917 | 26 | 9 | 0,171352 |
