# Supplementary material for: Evaluation of Orofacial and General Pain Location in Patients With Temporomandibular Joint Disorder—Myofascial Pain With Referral
Source: Front Neurol. 2019 May 29;10:546. doi: 10.3389/fneur.2019.00546 (PMC6549135; doi:10.3389/fneur.2019.00546)

1. Cervical spine


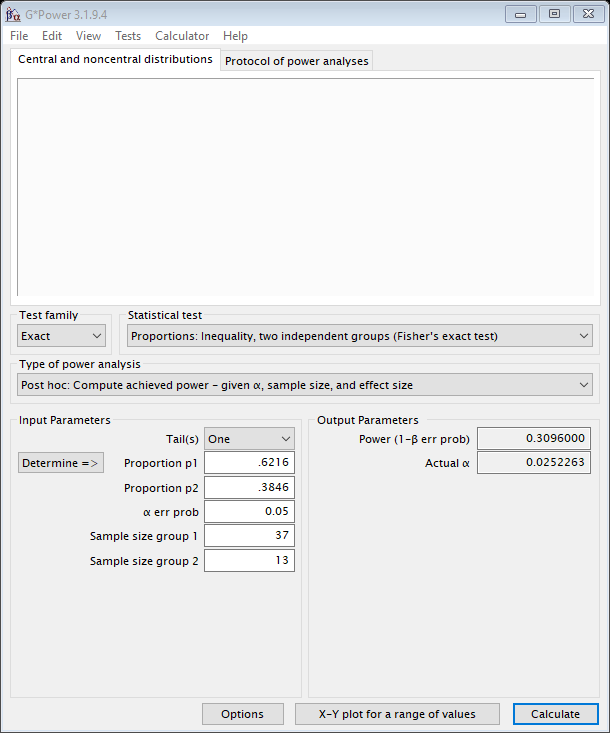


2. Thoracic spine Tx


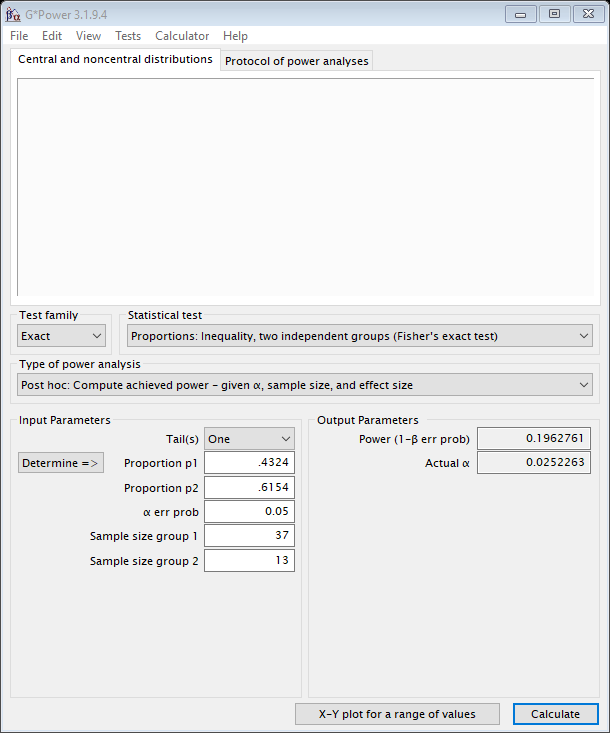


3. Lumbar spine Lx


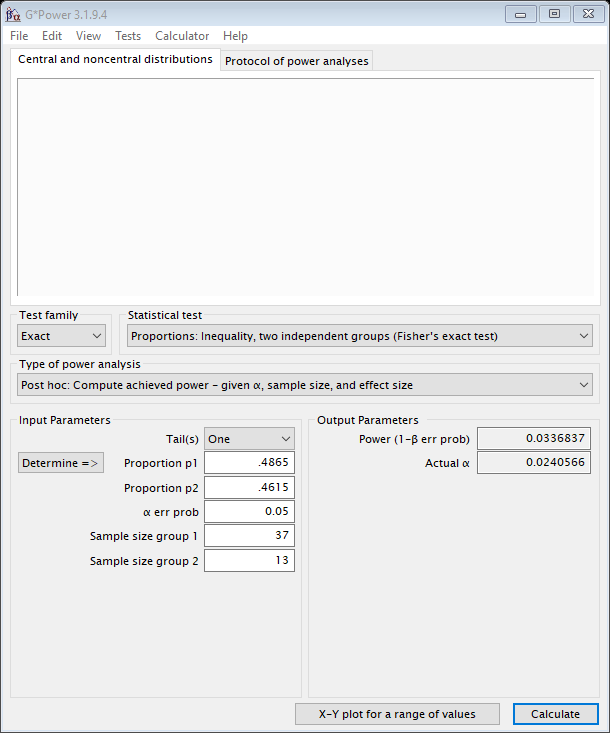


4. Sacrum Sc


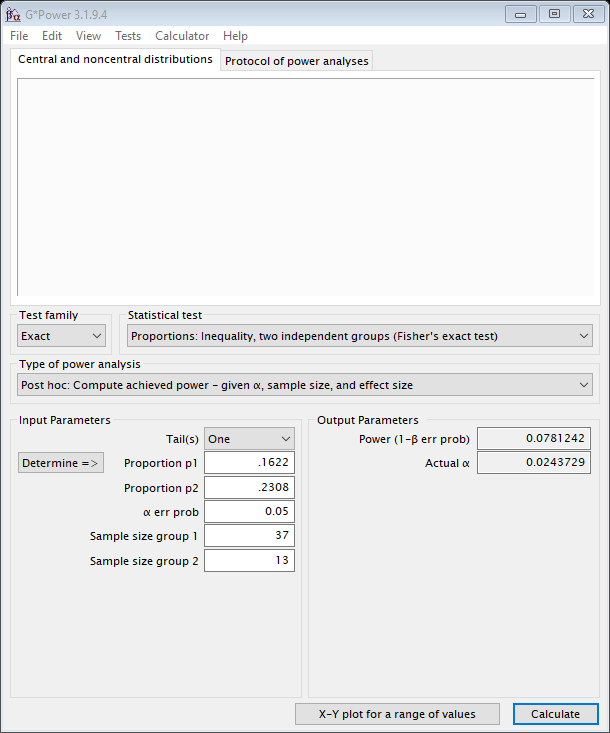


5. Pelvis


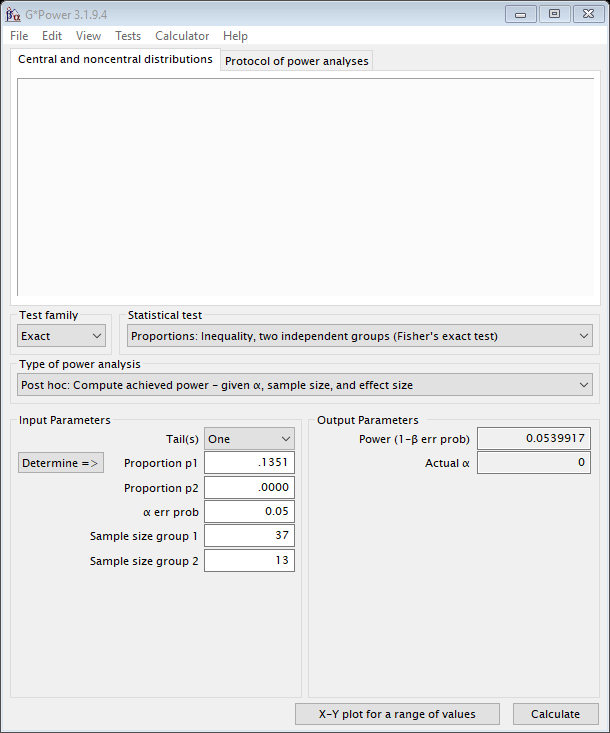


6. Temporal muscle on the right side


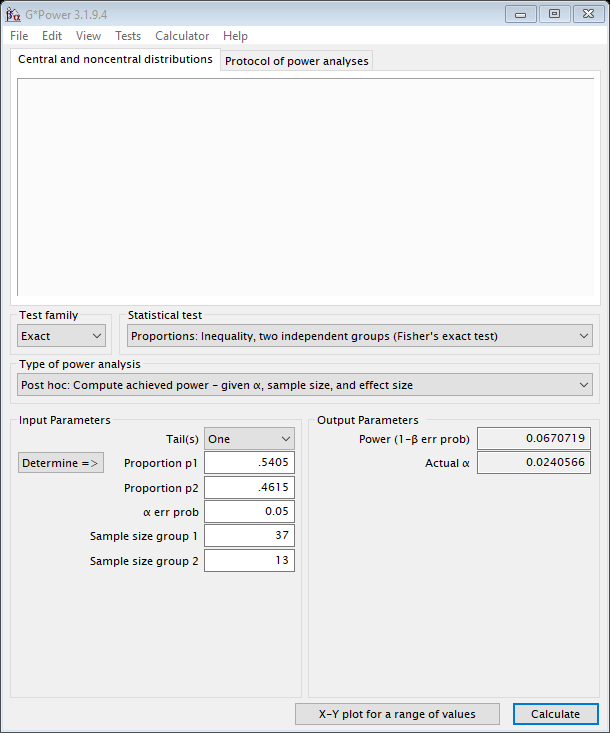


7. Temporal muscle on the left side


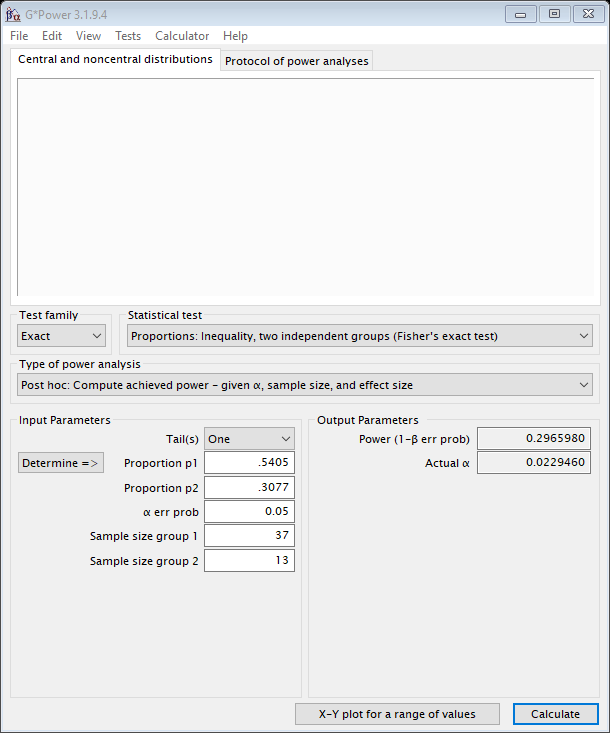


8.Masseter muscle on the right side


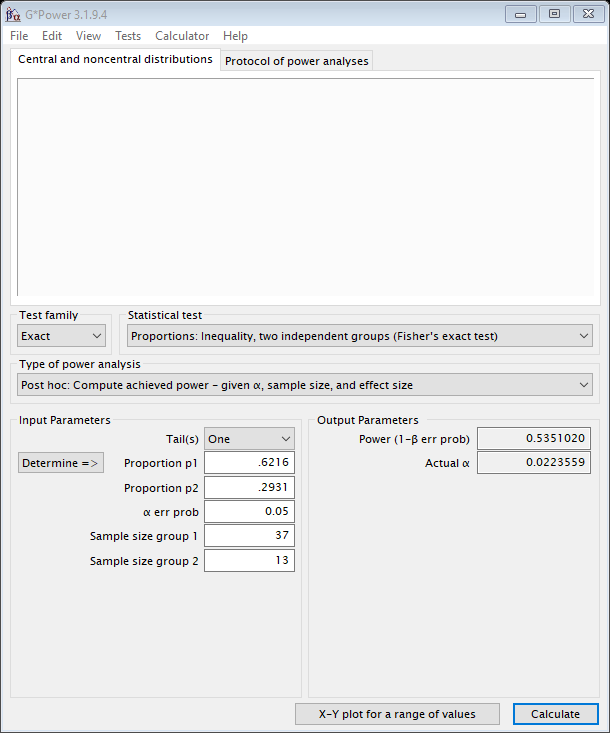


9. Masseter muscle on the left side


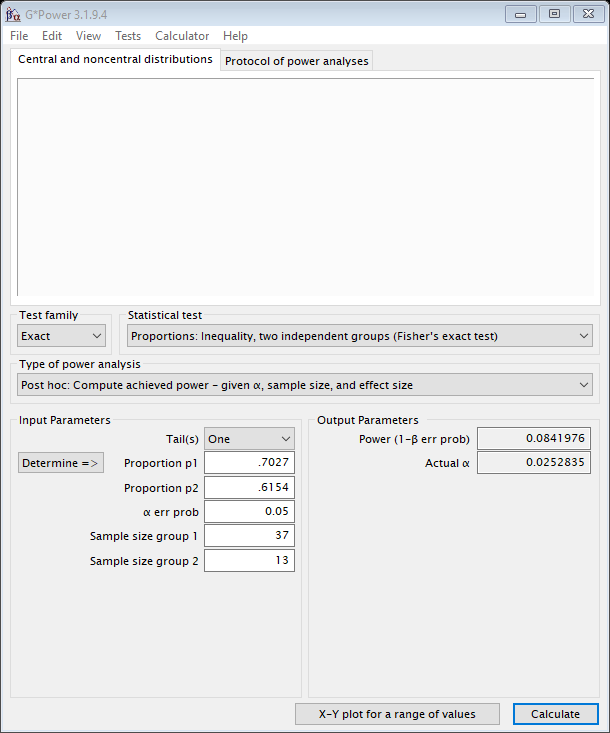


10. Musculus sternocleidomastoideus on the right side


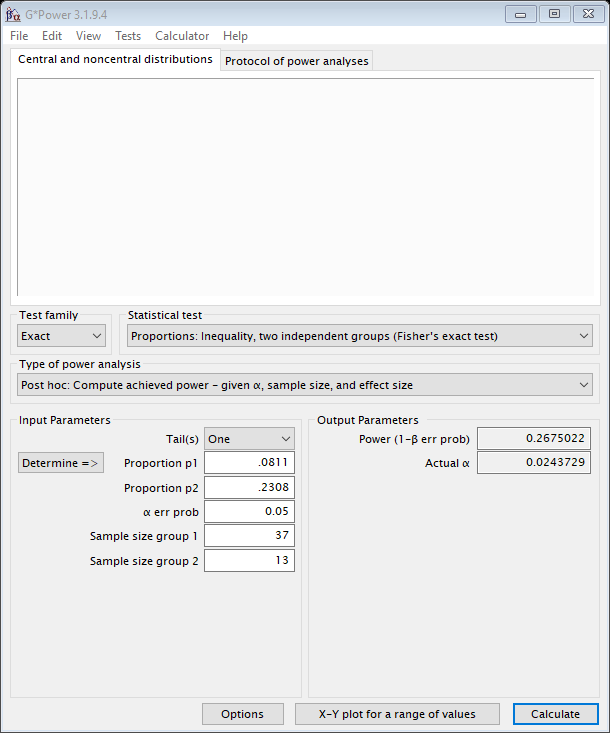


11. Musculus sternocleidomastoideus on the left side


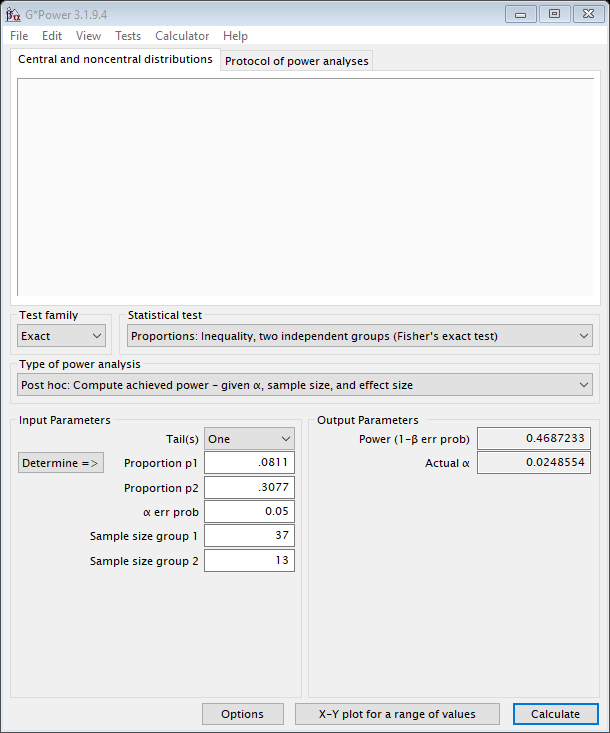


12. TMJ on the left side


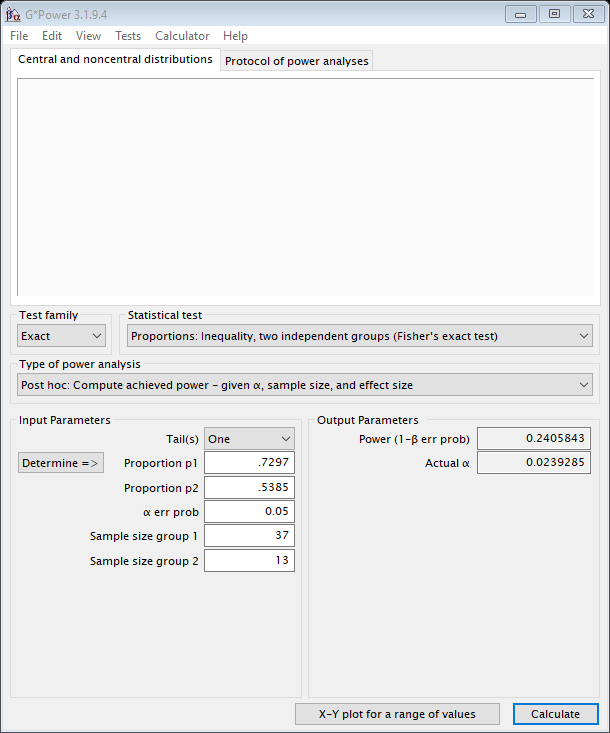


13. TMJ on the right side


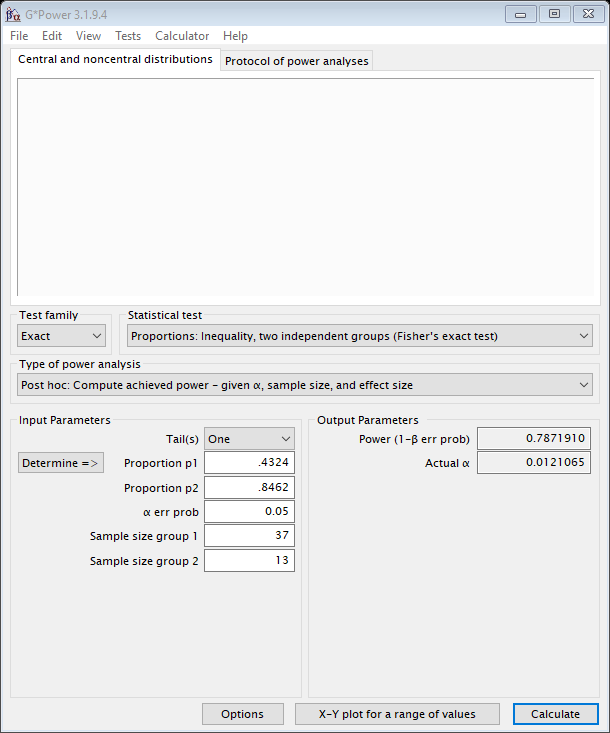


14. Area of the lower angle of the left scapula


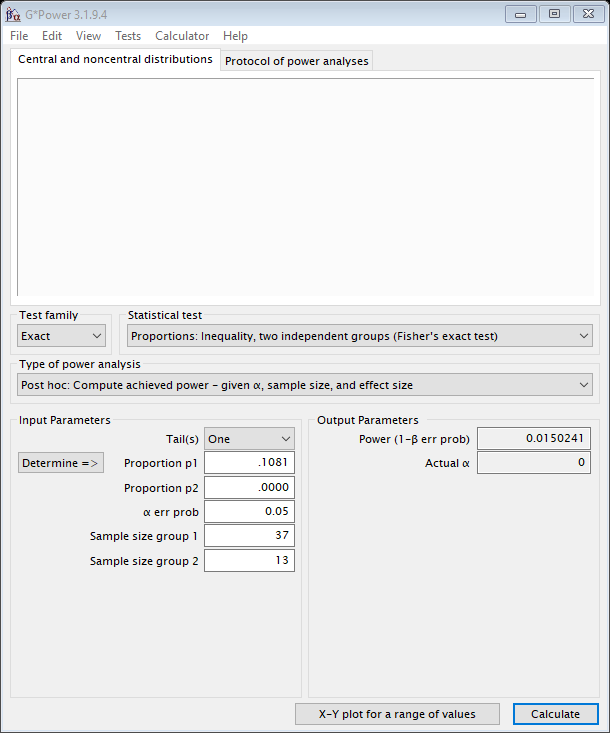


15. Area of the lower angle of the right scapula


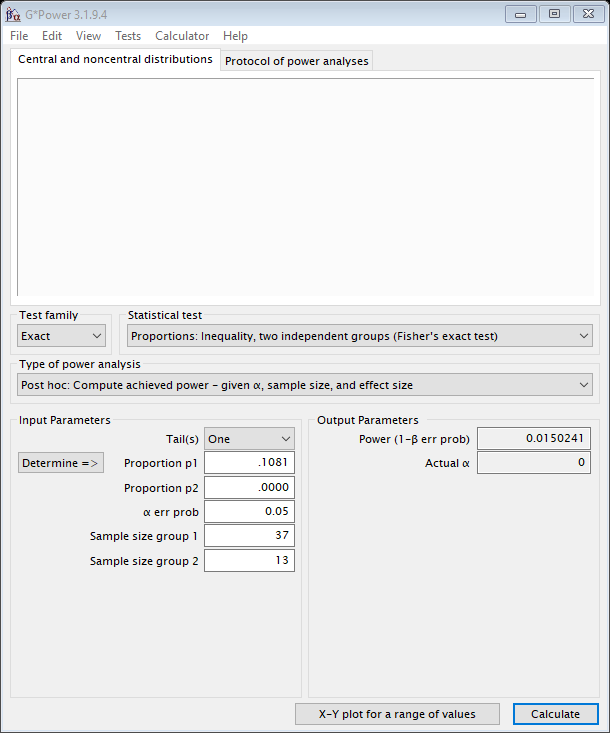


16. Right shoulder


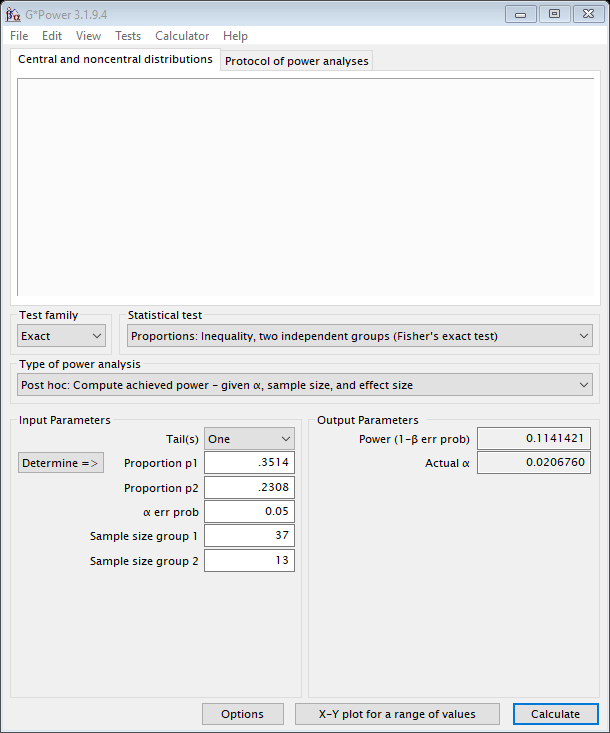


17. Left shoulder


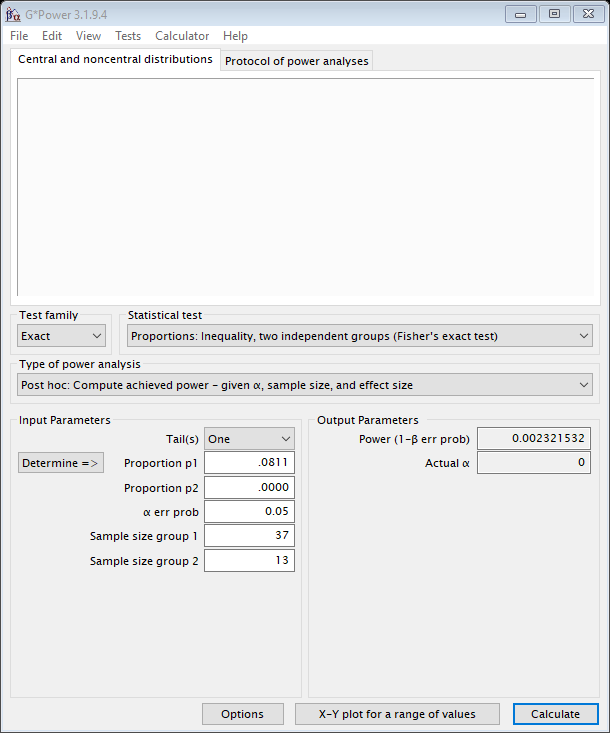


18. Thoracic outlet


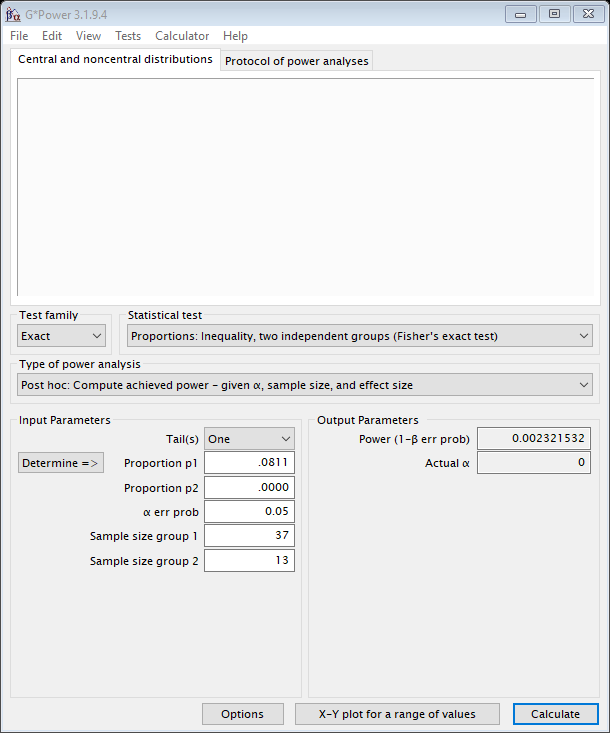


19. Right hip band


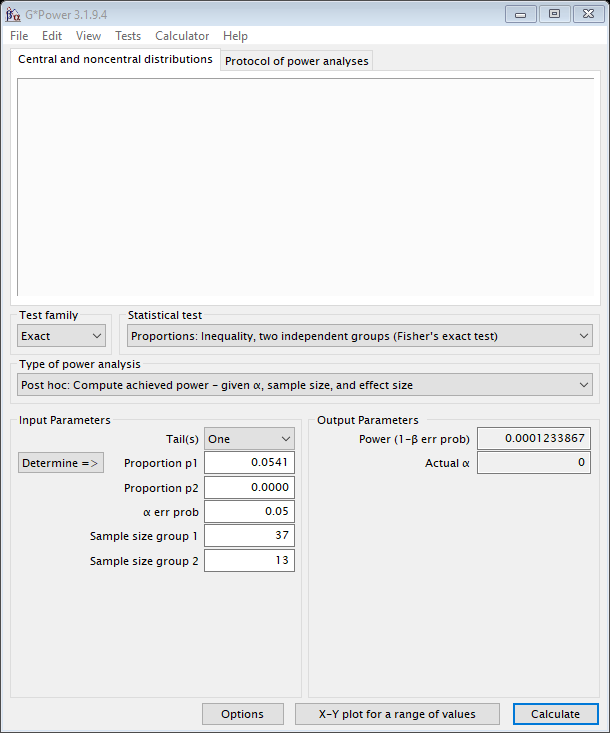


20. Left hip band


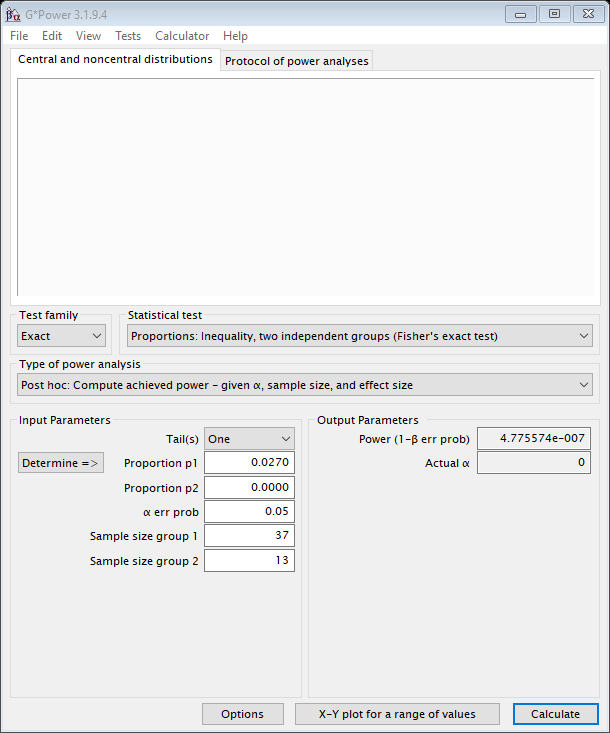


21. Sternum


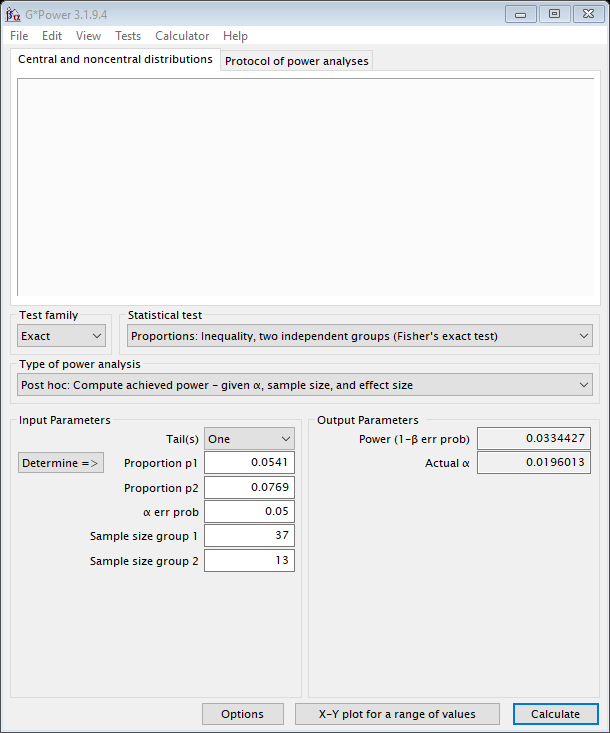


22. Right knee


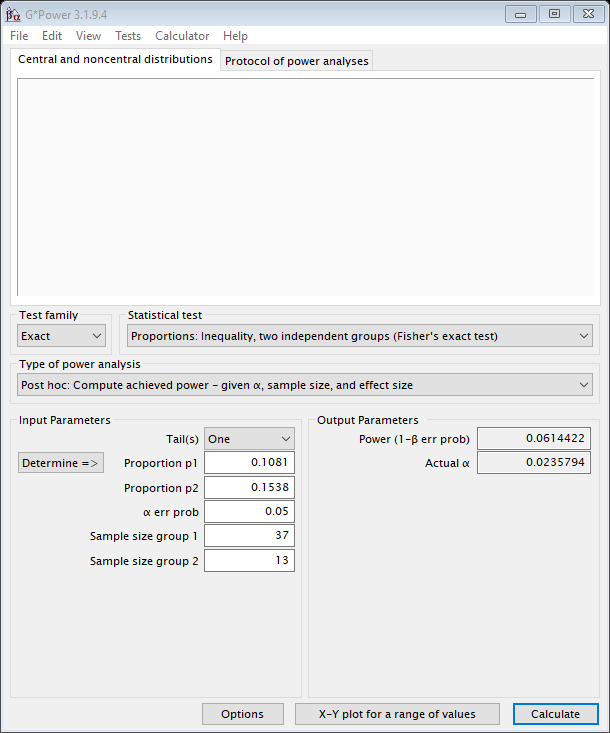


23. Left knee


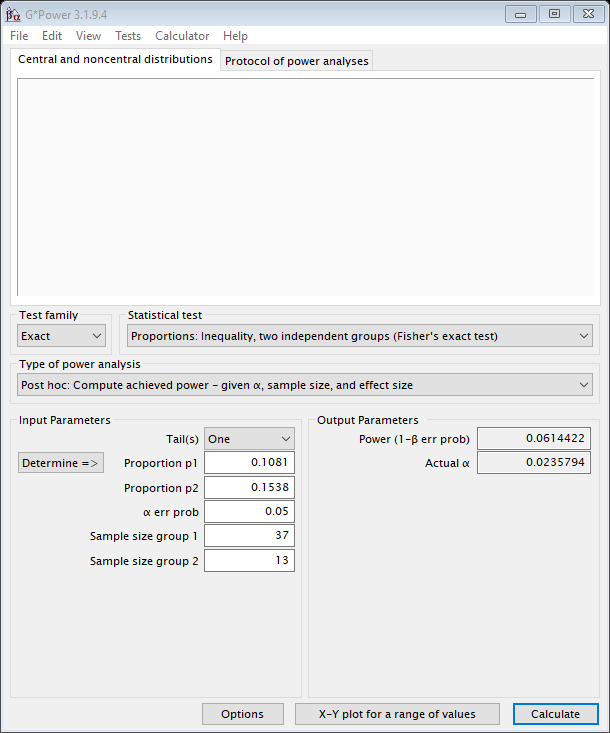


24. Right shinbone


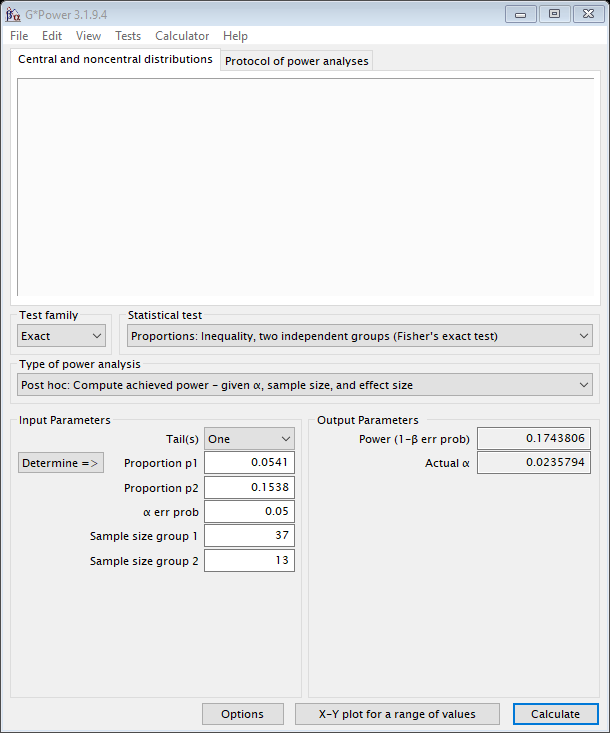


25. Left shinbone


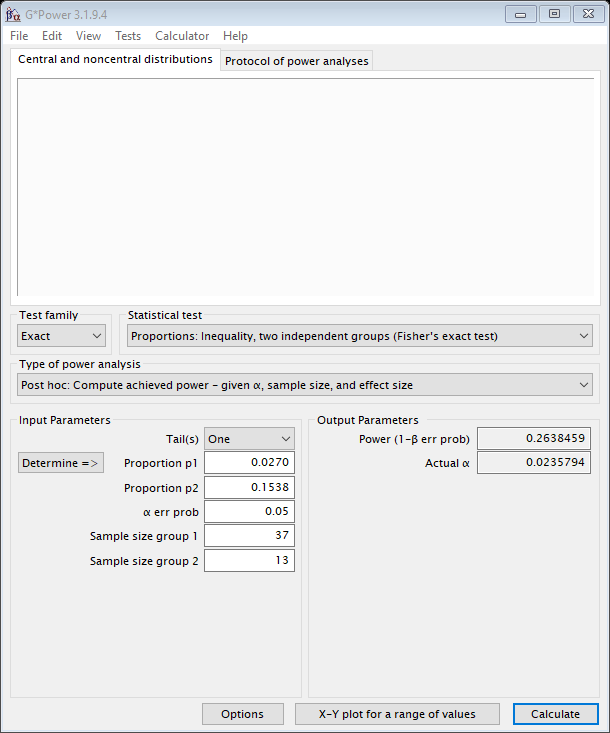


26. Teeth 18-14


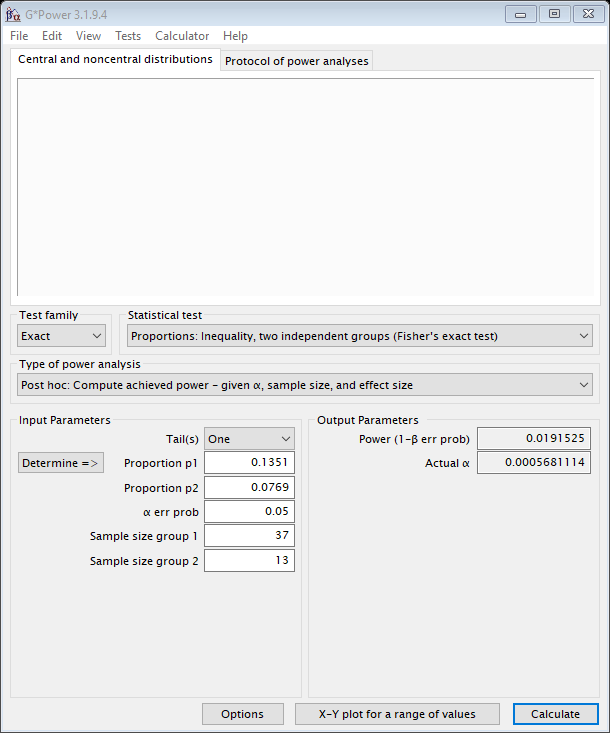


27. Teeth 13-11


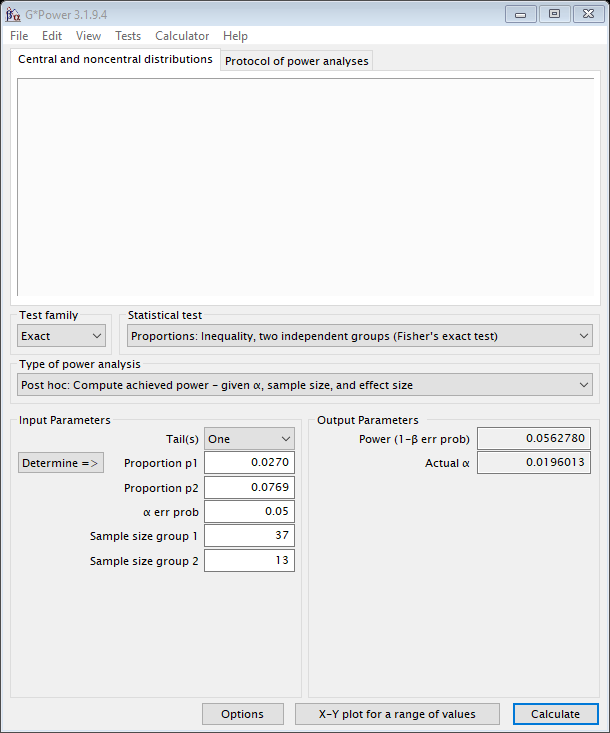


28. Teeth 21-23


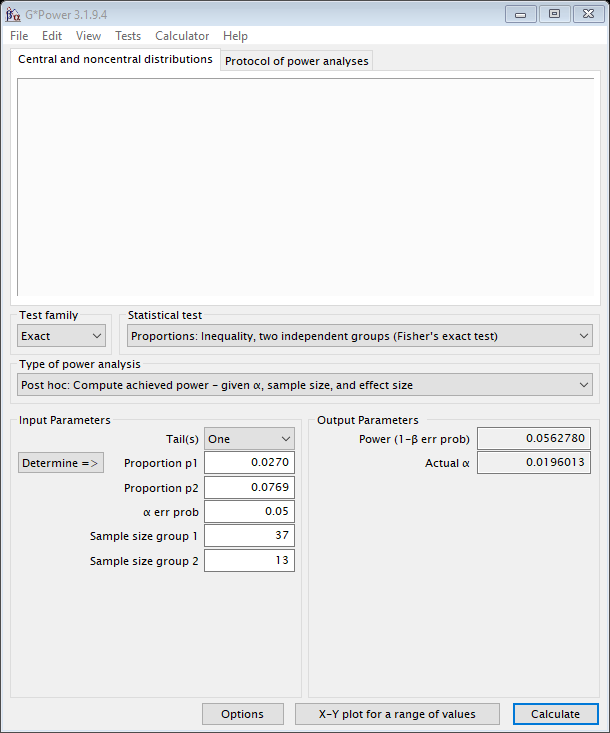


29. Teeth 24-28


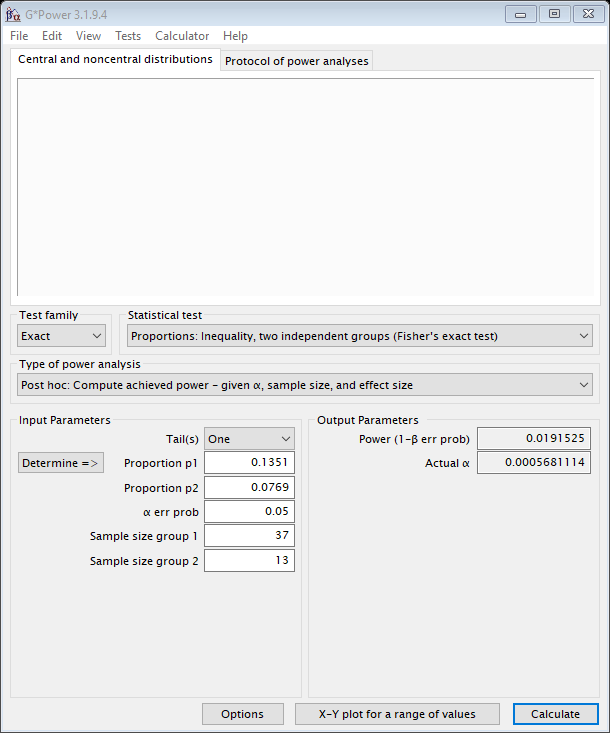


30. Teeth 34-38


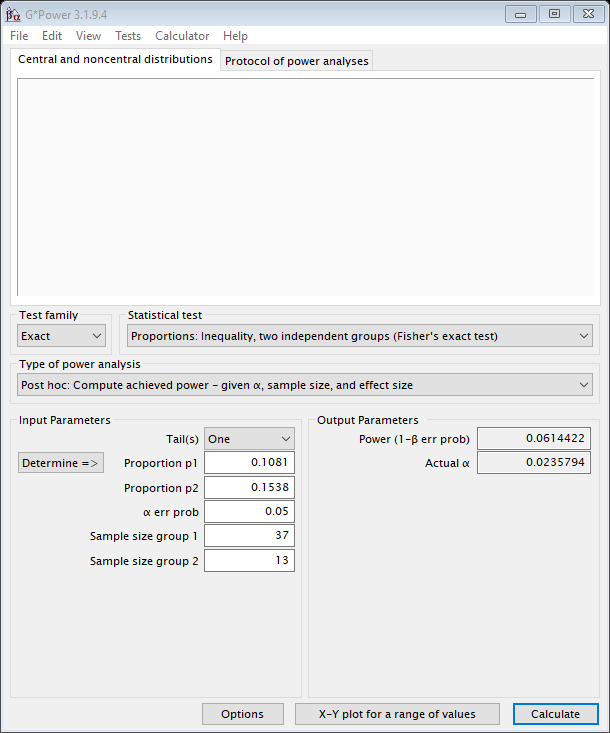


31. Teeth 31-33


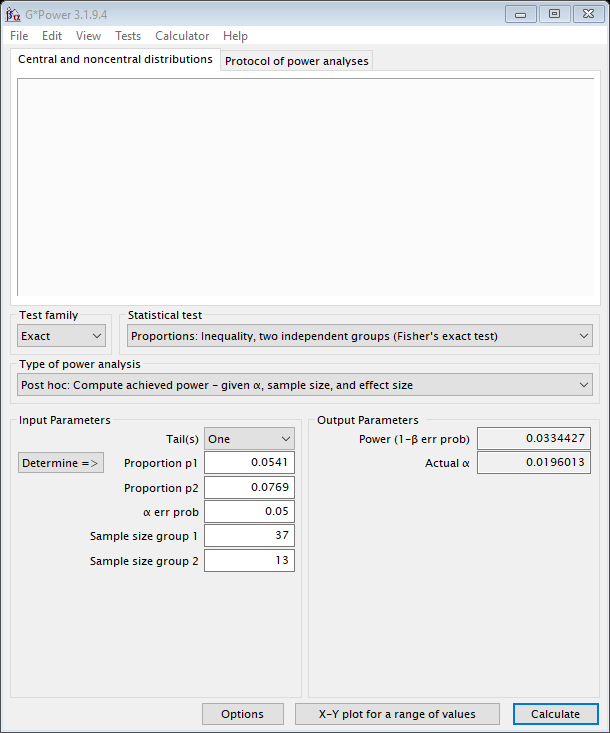


32. Teeth 41-43


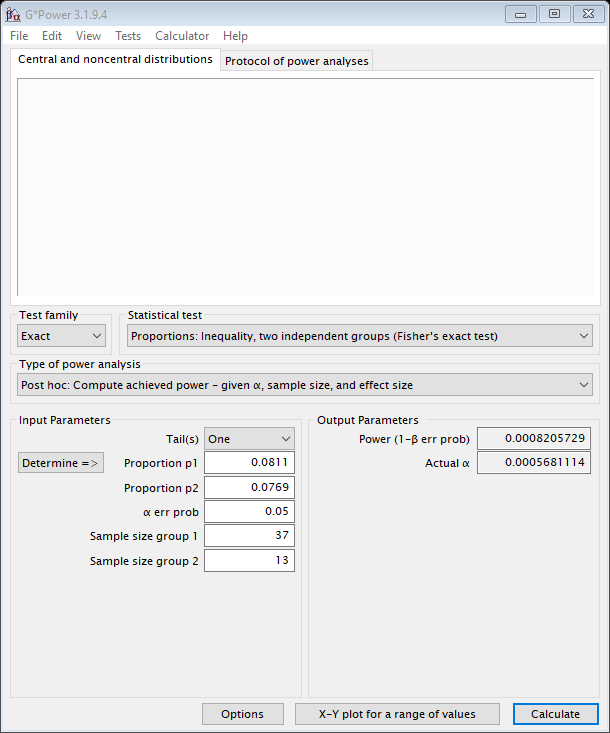


33. Teeth 44-48


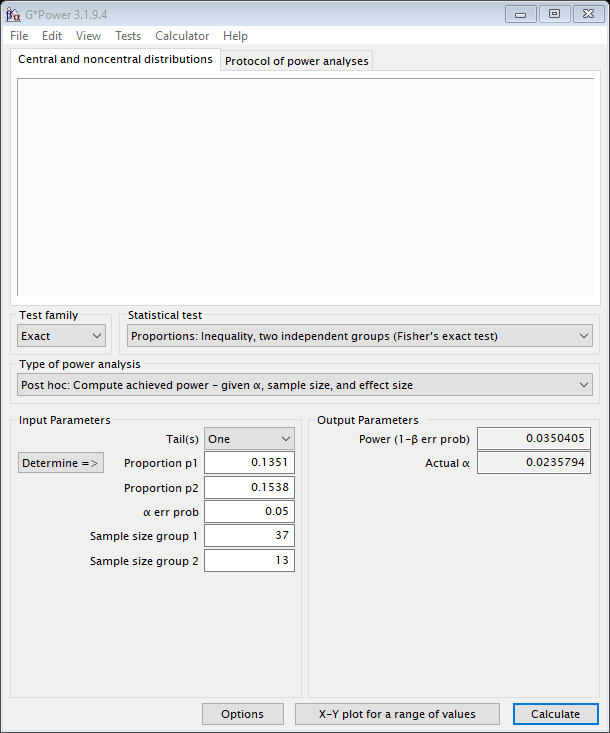


34. Palate


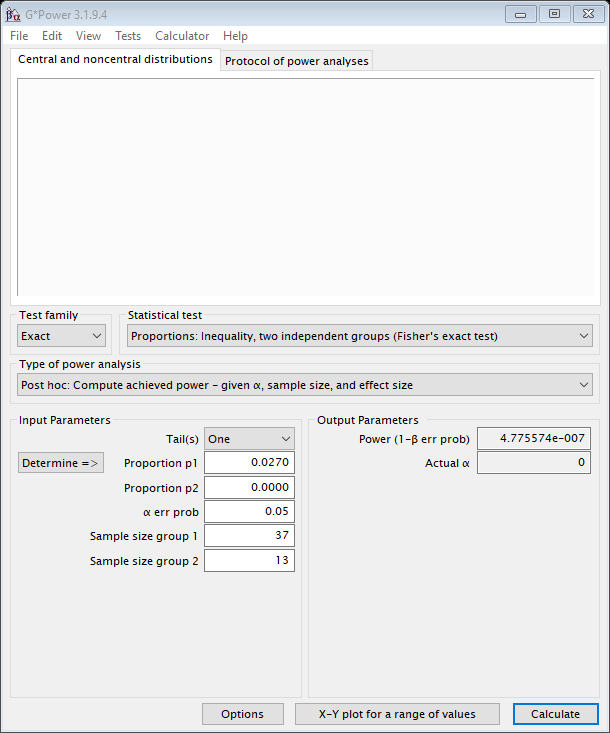


35. Right hand


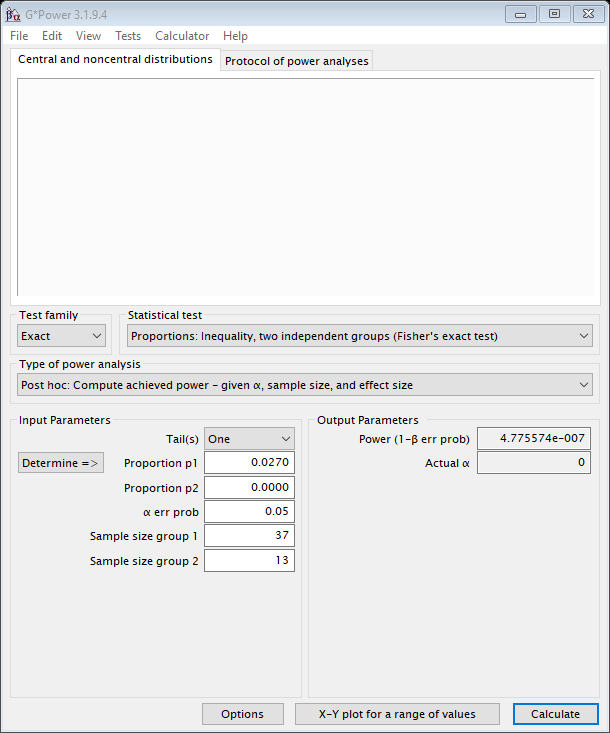


36. Left hand


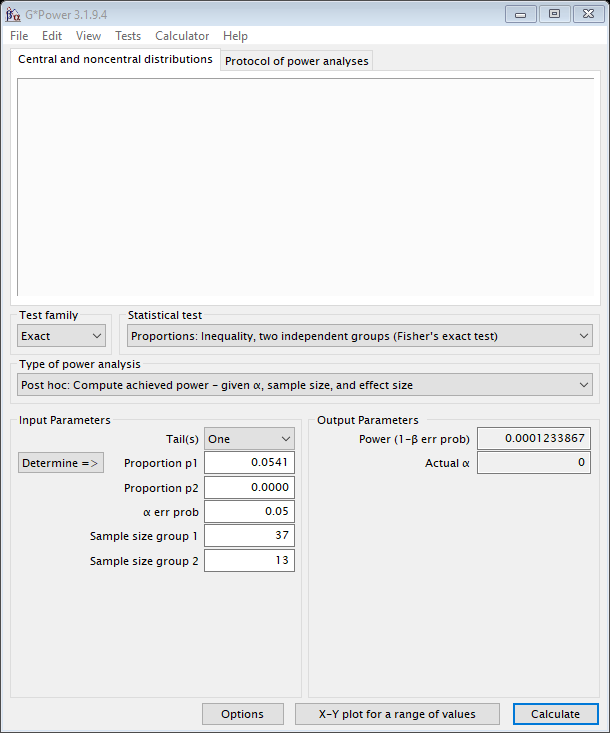


37. Right pterygomandibular ligament


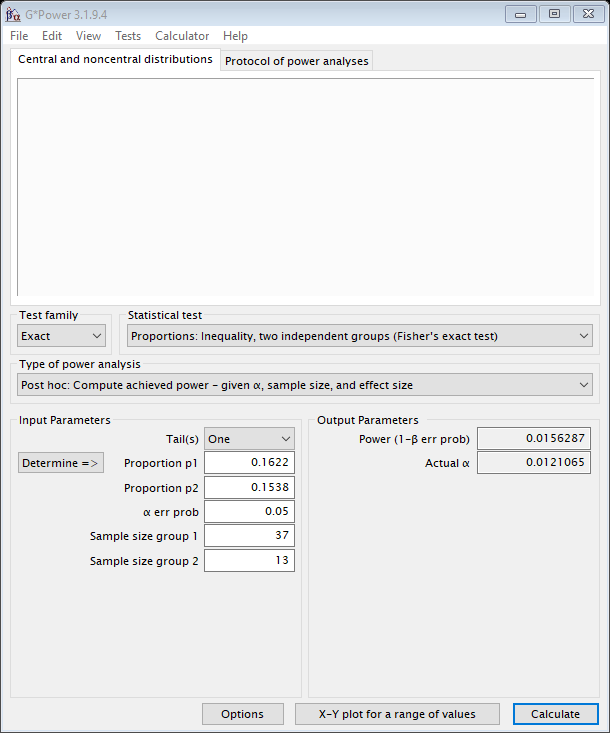


38. Left pterygomandibular ligament


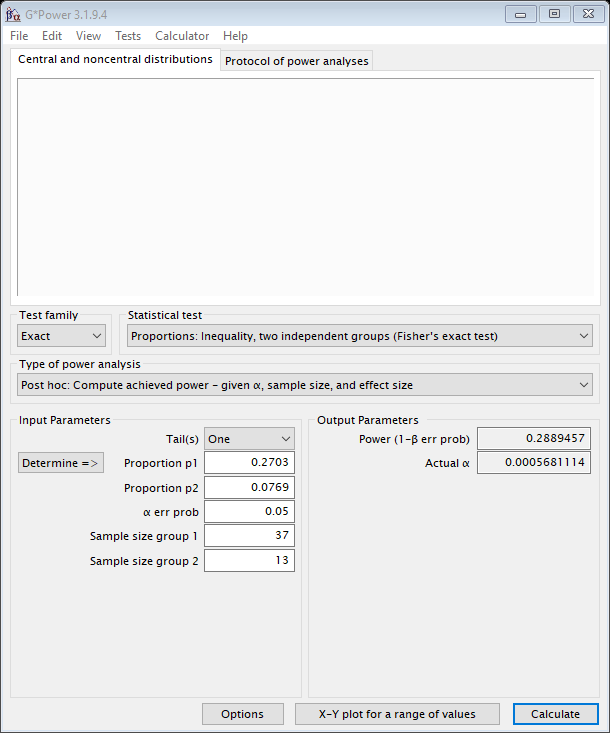


39. Right eye


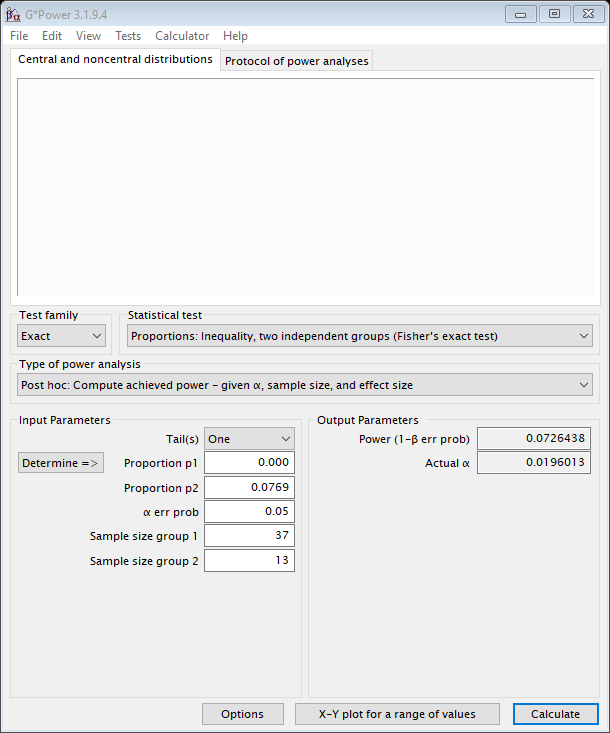


40. Left eye


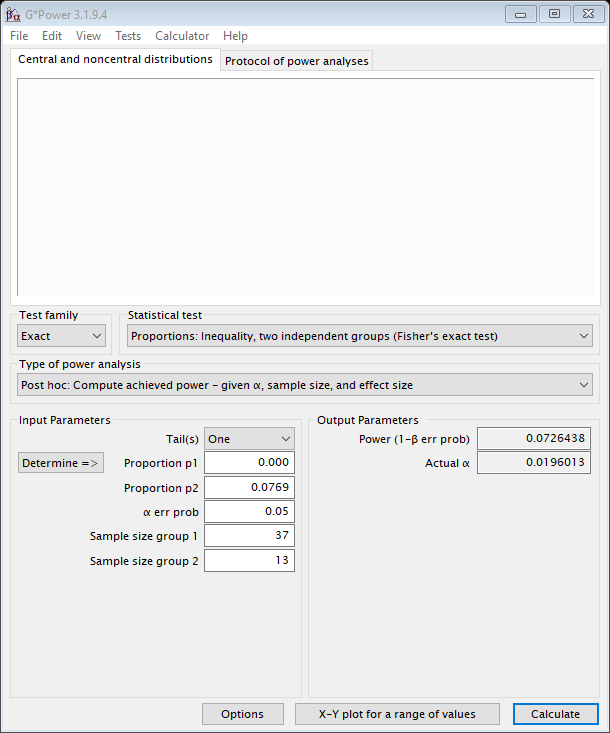

Supplement: Supplementary file 2 [file Data_Sheet_2.docx]
